# Supplementary figures and images for: Inference of coevolutionary dynamics and parameters from host and parasite polymorphism data of repeated experiments
Source: PLoS Comput Biol. 2020 Mar 23;16(3):e1007668. doi: 10.1371/journal.pcbi.1007668 (PMC7156111; doi:10.1371/journal.pcbi.1007668)

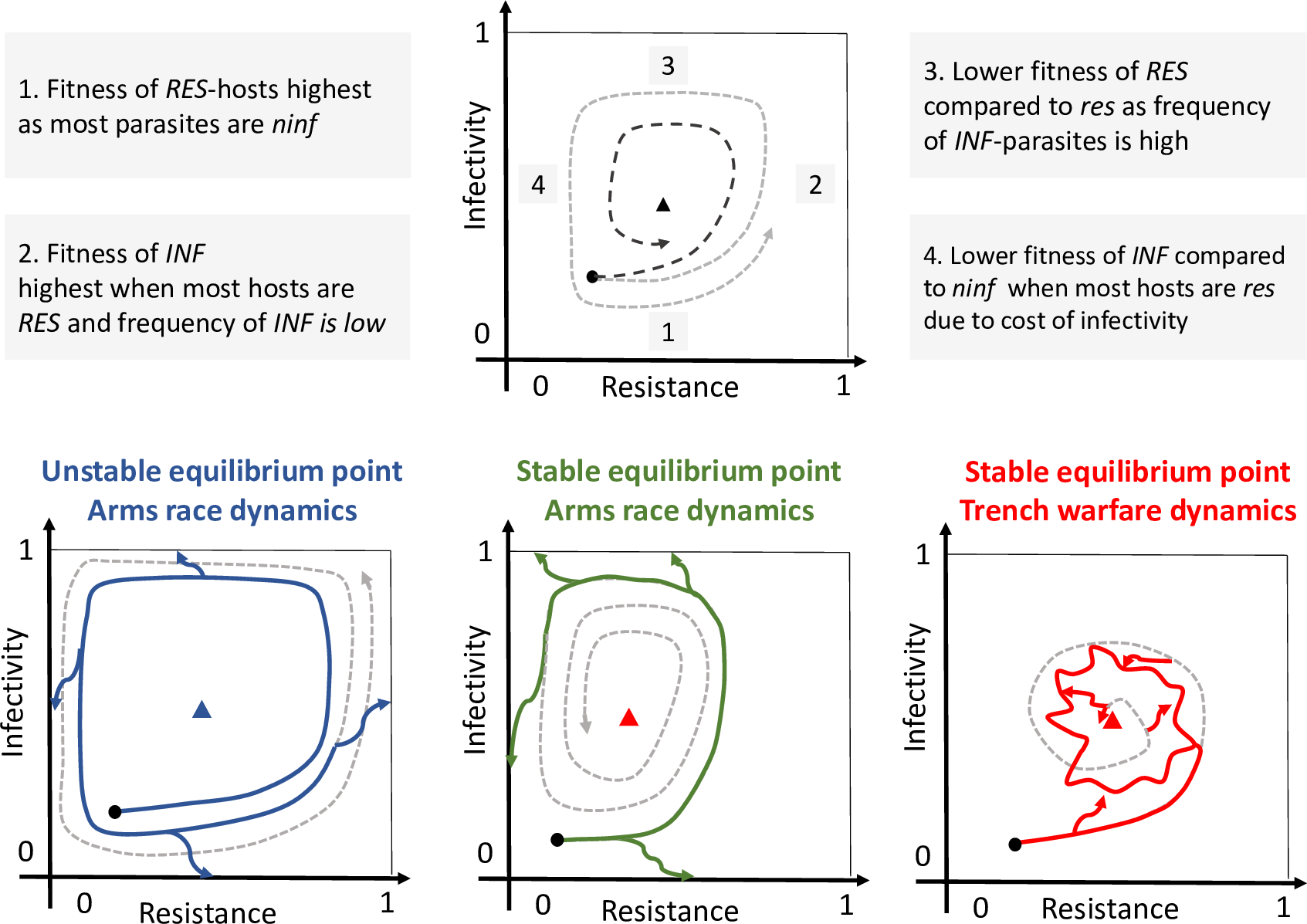

Supplement: S1 Fig — Schematic illustrating the evolutionary forces and interactions driving the coevolutionary cycles on top. The different coevolutionary dynamics in infinite population in interaction with genetic drift are shown on the bottom. The grey line always shows the expected dynamics in infinite population size over time. The frequency of the resistant allele in the host is always shown on the x-axis, the frequency of the infectivity allele in the parasite on the y-axis. The triangles are the unstable/stable internal equilibrium points of the model. The effect of genetic drift on the allele frequency path due to finite population size is always shown in color. Bottom left: Model with arms-race dynamics in infinite and finite population size. Bottom middle: Model with trench-warfare dynamics in infinite population size and arms-race dynamics in finite population size. Bottom right: Model with trench-warfare dynamics in infinite and finite population size. (TIF) [file pcbi.1007668.s001.tif]

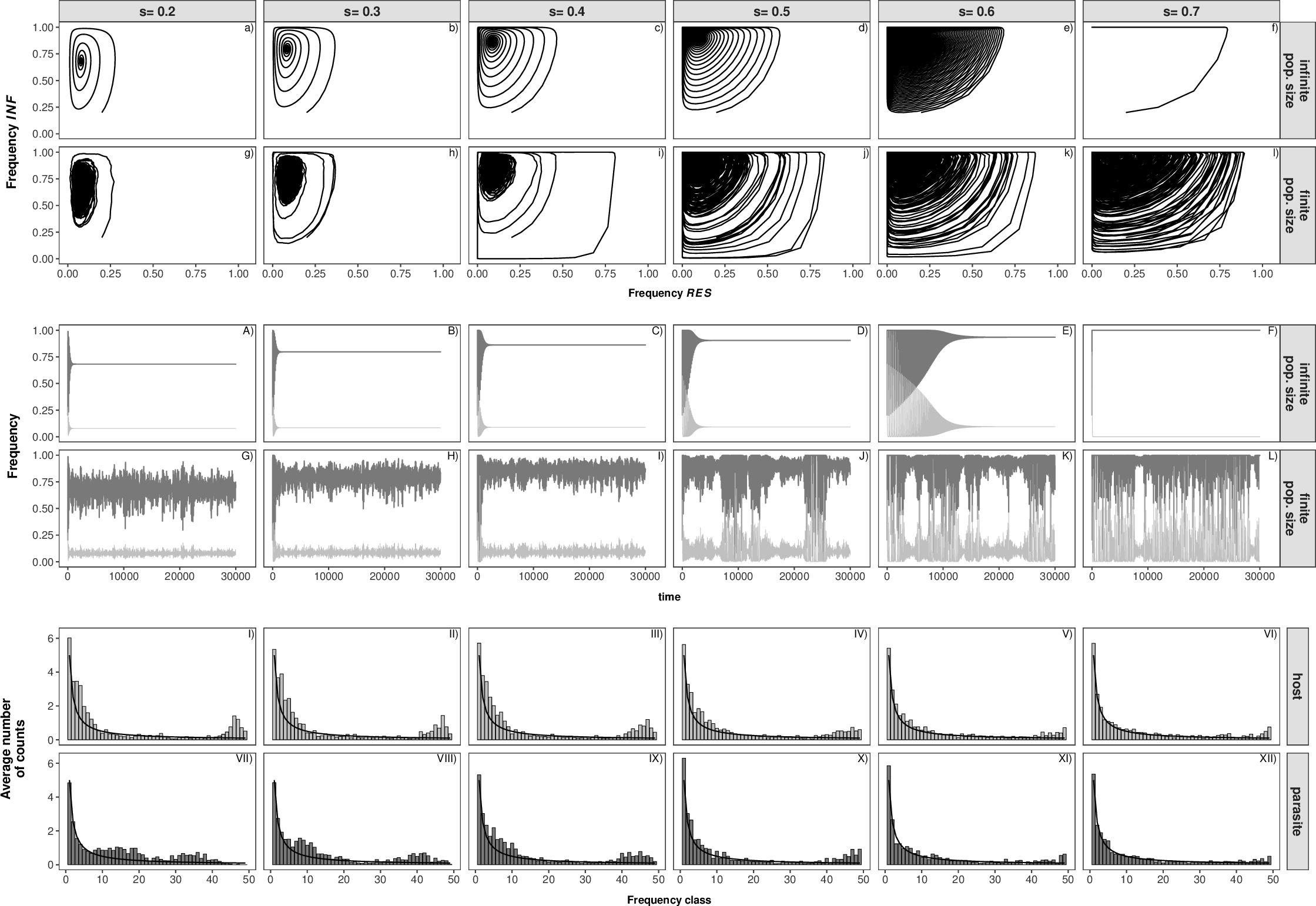

Supplement: S2 Fig — Influence of the cost of infection (s) on the coevolutionary dynamics and genetic signatures in Model A. The subfigures show the allele frequency trajectory in infinite population size (a-f, A-F), one exemplary allele frequency path in finite population size which takes genetic drift and functional mutations into account (d-f, D-F), the average unfolded host site frequency spectrum of r = 200 repetitions (I-VI) and the average unfolded parasite site frequency spectrum of r = 200 repetitions (VII-XII). In subfigures a-l each dot represents the frequency of resistant (RES) hosts (x-axis) and infective (INF) parasites (y-axis) at the beginning of a single host generation g. The same information is displayed in a slightly different way in subfigures A-L. Here, the frequencies of resistant (RES) hosts (light grey) and infective (INF) parasites (dark grey) (y-axis) are plotted over time (x-axis). Costs are fixed to cH = 0.05, cP = 0.1. The results in finite population size are plotted for NH = NP = 10, 000, μRtor = μntoI = μrtoR = μIton = 10−5. The site frequency spectra are shown for θP = θH = 5 and nH = nP = 50. (TIF) [file pcbi.1007668.s002.tif]

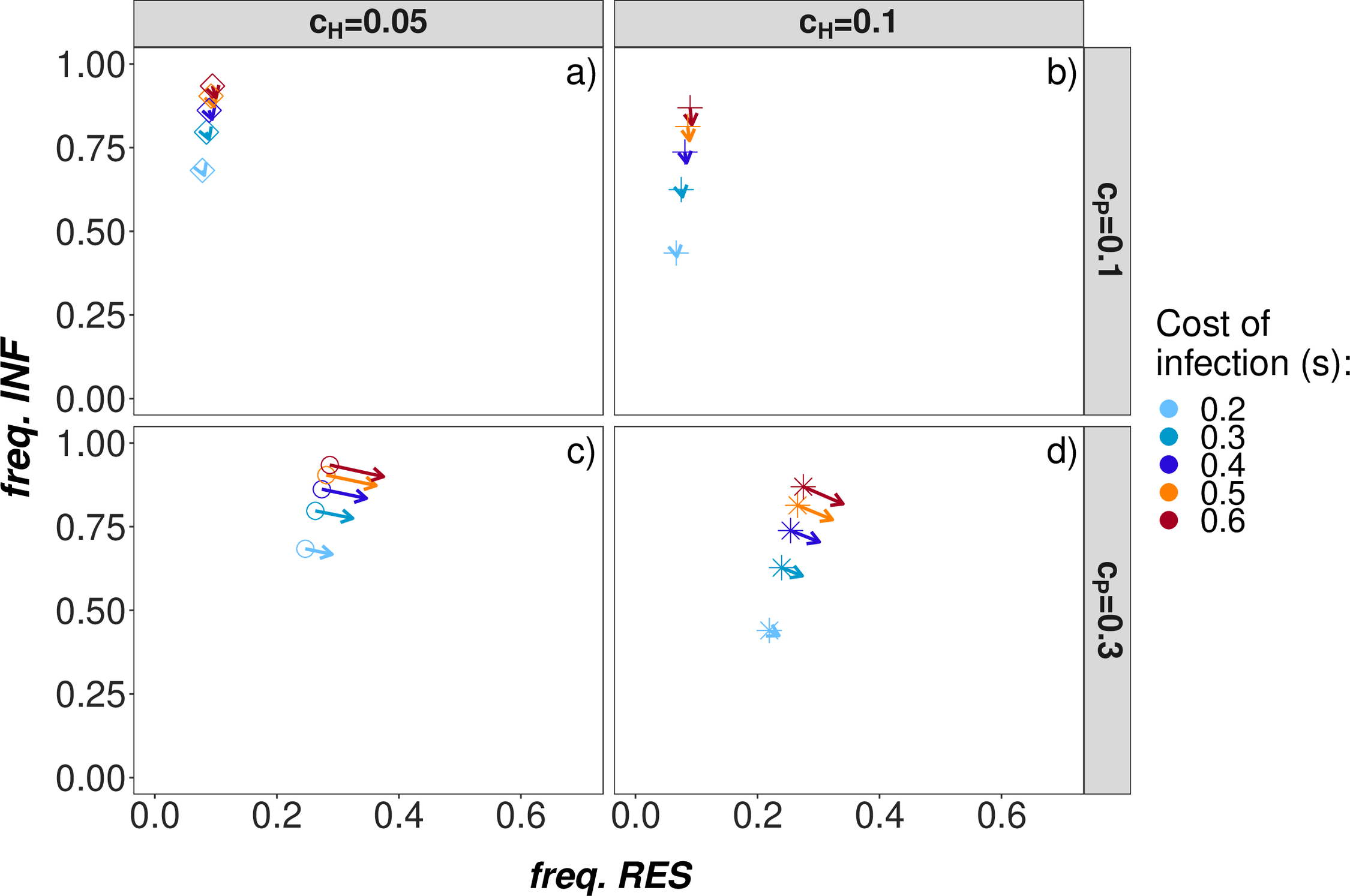

Supplement: S3 Fig — Deterministic equilibrium frequencies for model A for different combinations of cost of resistance cH = (0.05, 0.1) (columns), cost of infectivity cP = (0.1, 0.3) (rows) and cost of infection s = (0.2, 0.3, 0.4, 0.5, 0.6, 0.7, 0.8) (color of the squares). Only parameter combinations with trench-warfare dynamics are shown. Centres of the dots represent the stable equilbrium frequencies obtained by simulating numerically the recursion equations Eq (2) for 30,000 generations starting with an initial frequency of R0 = 0.2 resistant hosts and a0 = 0.2 infective parasites. Heads of the arrows represent the equilibrium frequencies based on Eq (3) which slightly differ from the numerical computations due to analytical approximations. (TIF) [file pcbi.1007668.s003.tif]

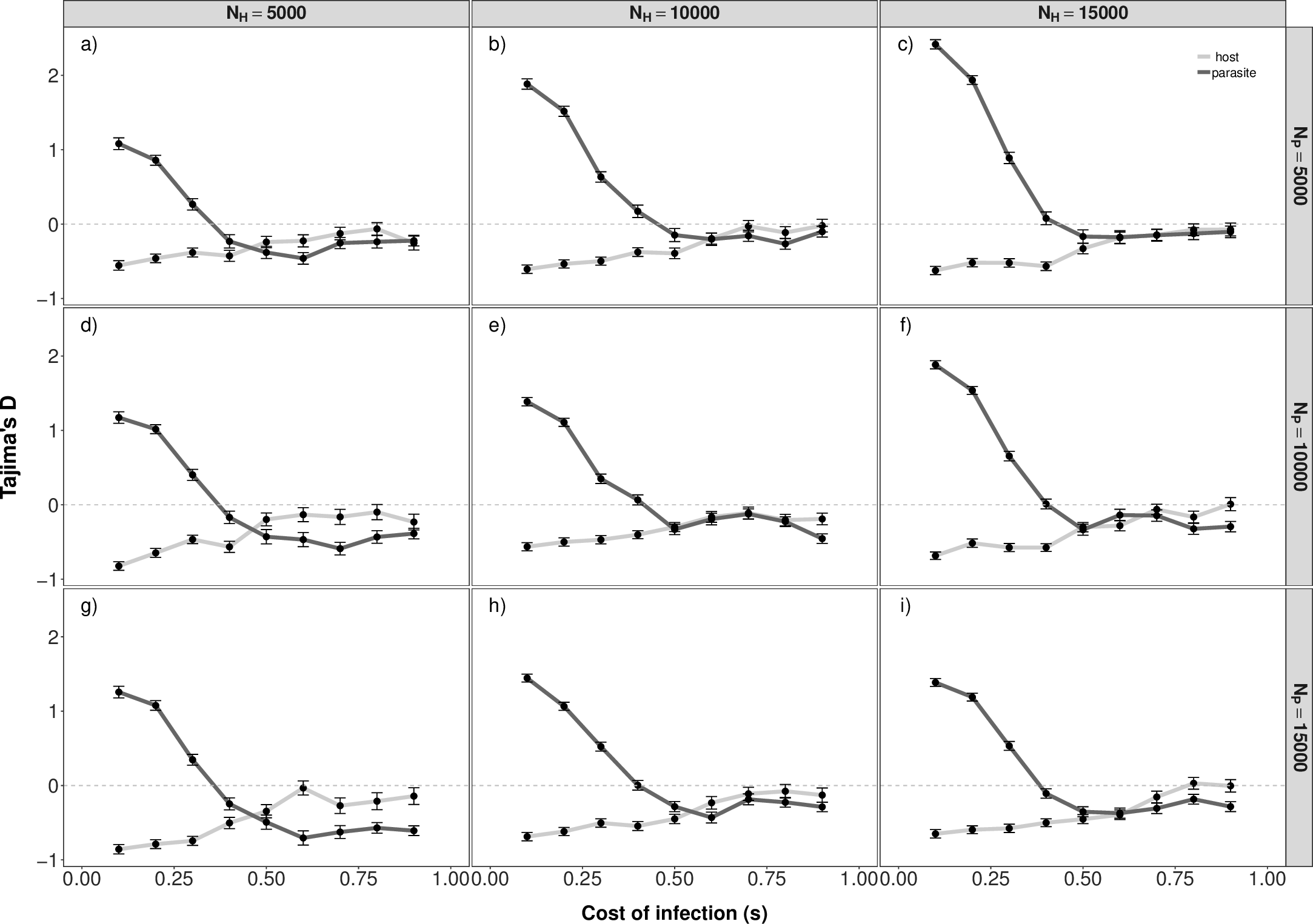

Supplement: S4 Fig — Tajima’s D (y-axis) for Model A for various cost of infection s (x-axis) and different combinations of NP (NP = 5, 000 top, NP = 10, 000 middle, NP = 15, 000 bottom) and NH (NH = 5, 000 left, NH = 10, 000 middle, NH = 15, 000 right). The mean and standard error of Tajima’s D of the parasite population (dark grey) and of the host population (light grey) are plotted for r = 200 repetitions. Note that subfigure e corresponds to S9a Fig. The other parameters are fixed to: cH = 0.05, cP = 0.1, θH = NH/2000, θP = NP/2000, nH = nP = 50, μRtor = μrtoR = μntoI = μIton = 10−5. (TIF) [file pcbi.1007668.s004.tif]

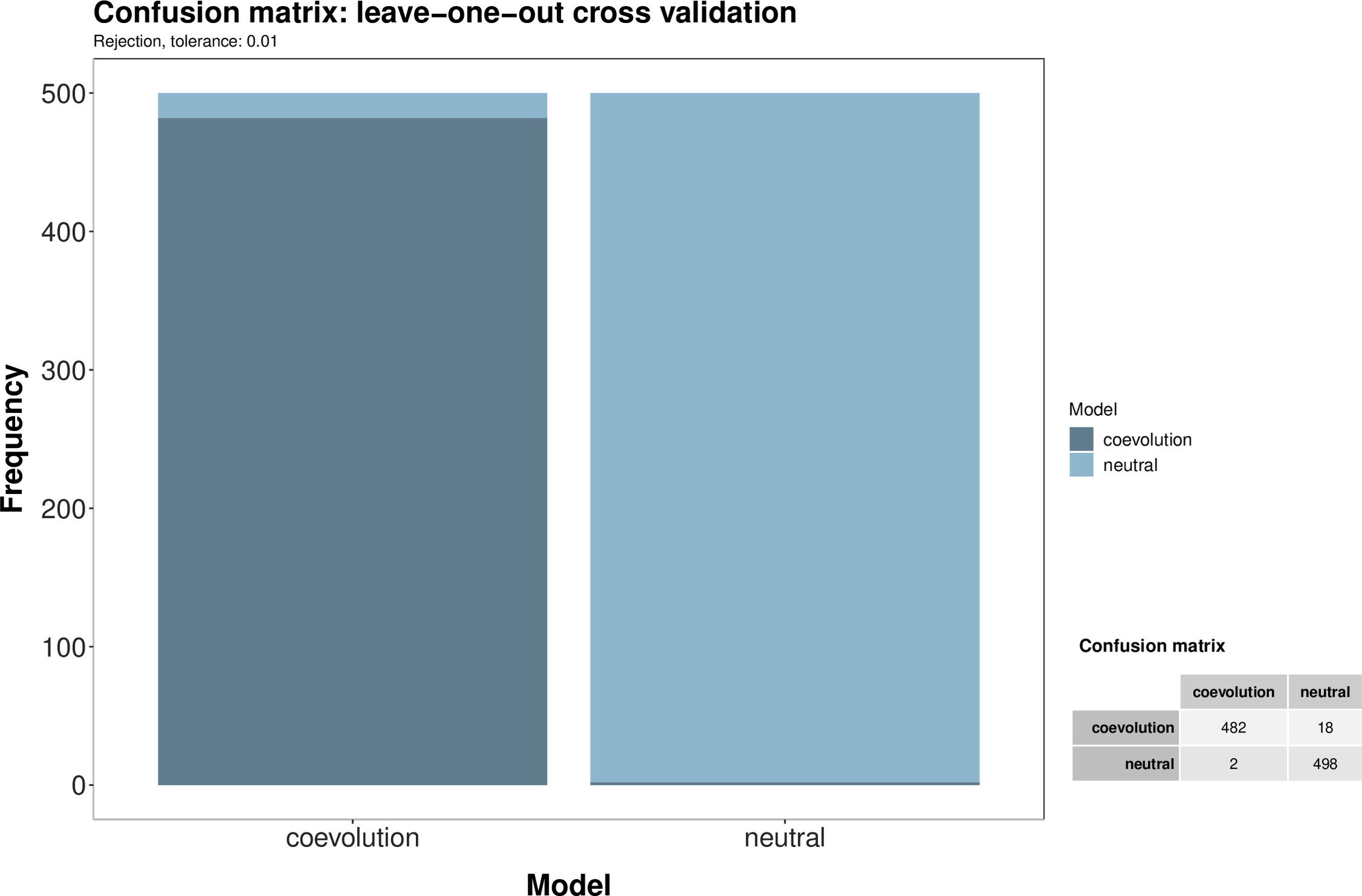

Supplement: S5 Fig — Leave-one-out-cross-validation result for distinguishing the coevolution model with unknown costs of infection (s), host population size (NH) and parasite population size (NP) from a neutral model with a unknown host and parasite population sizes. Cross-validation results are shown for r = 30 and are based on 500 randomly chosen ABC-simulations for each model. (TIF) [file pcbi.1007668.s005.tif]

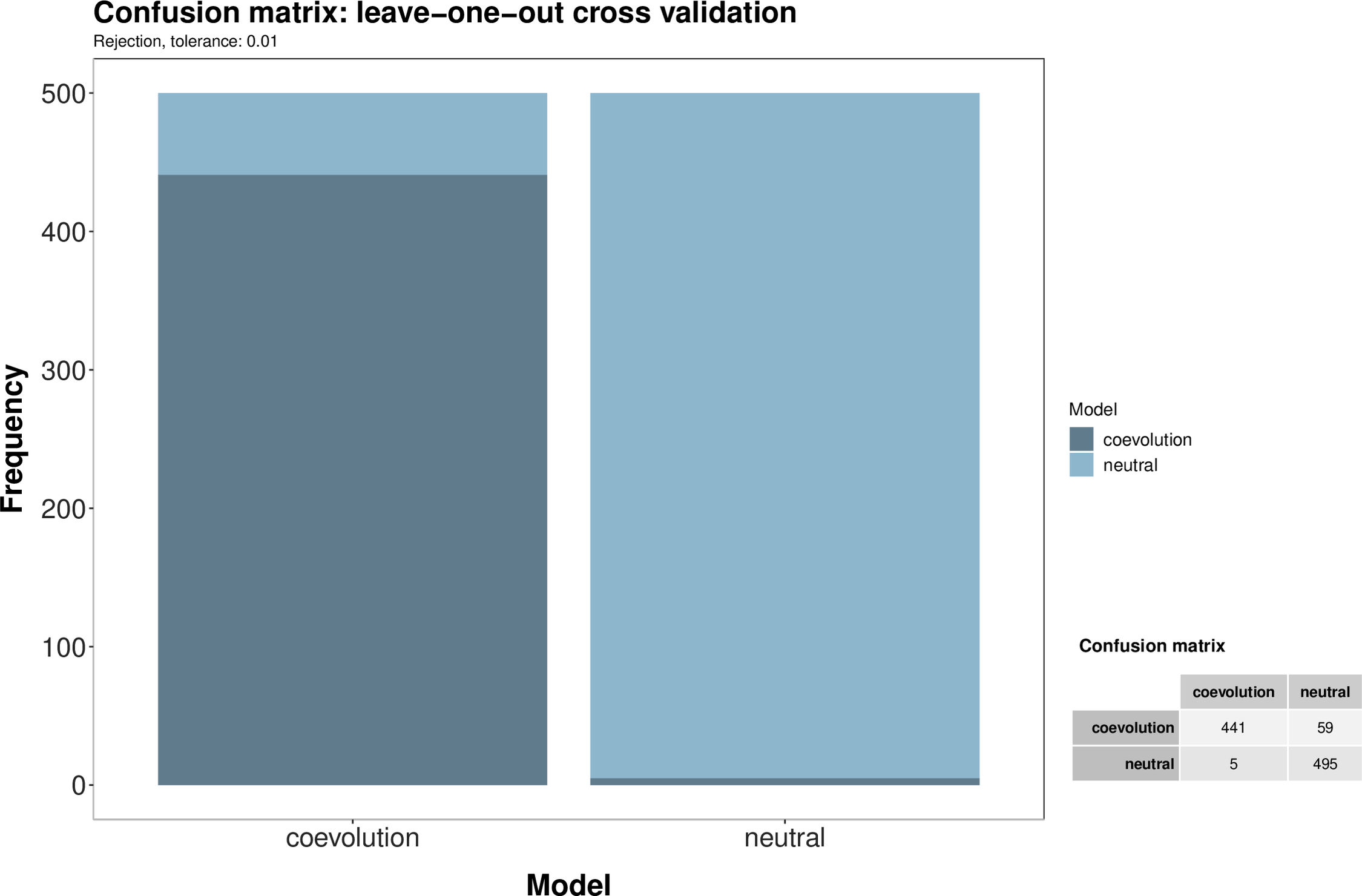

Supplement: S6 Fig — Leave-one-out-cross-validation result for distinguishing the coevolution model with unknown costs of infection (s), host population size (NH) and parasite population size (NP) from a neutral model with unknown host and parasite population sizes. Cross-validation results are shown for r = 10 and are based on 500 randomly chosen ABC-simulations for each model. (TIF) [file pcbi.1007668.s006.tif]

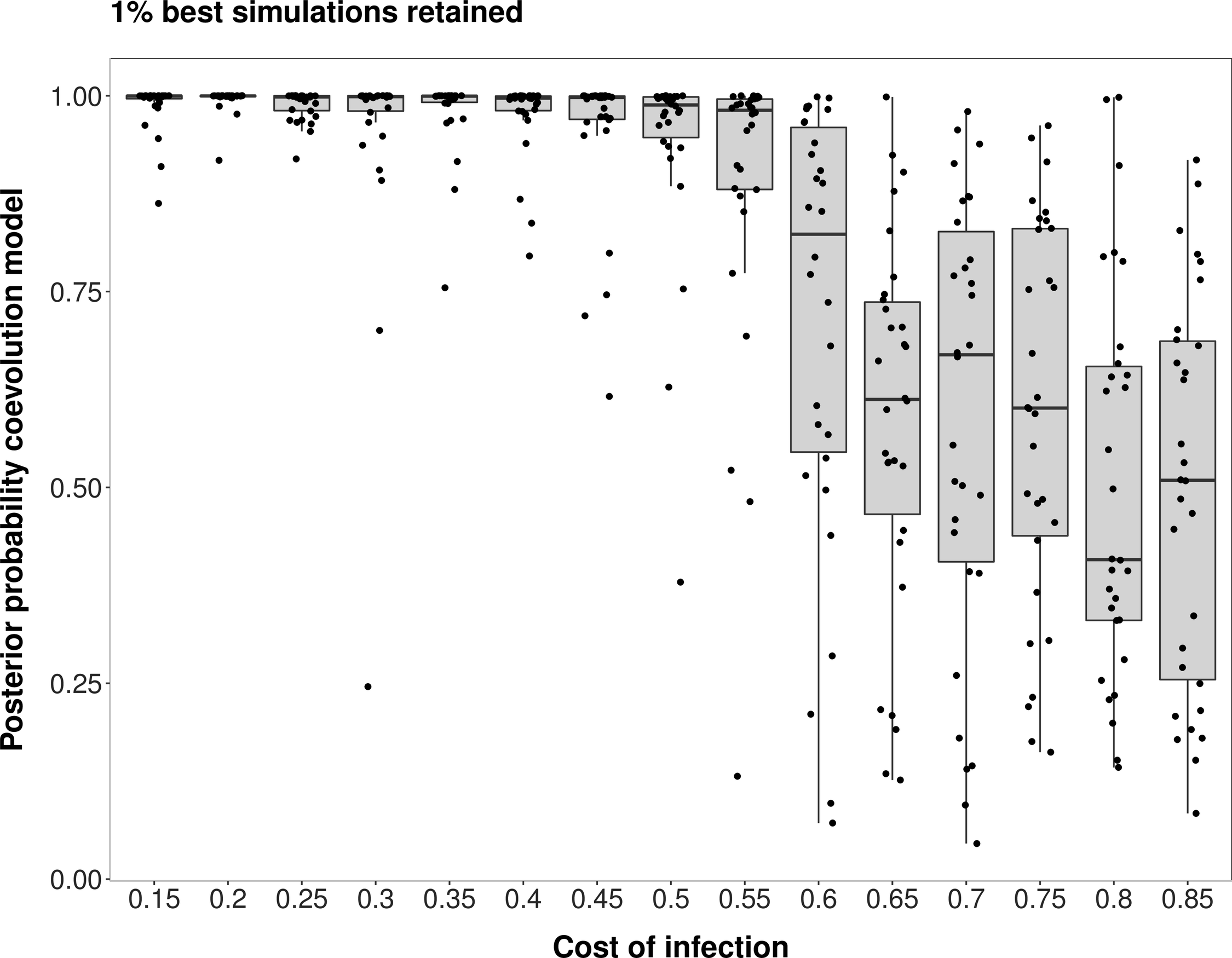

Supplement: S7 Fig — Model choice results for scenario 1 for r = 10. Model choice has been run to distinguish a coevolution model with unknown costs of infection (s), host population size (NH) and parasite population size (NP) from a neutral model with unknown host and parasite population sizes. Model choice is shown for r = 30 repetitions and based on the 1% simulations having the closest summary statistics to those of the PODs. The posterior probability in support of the coevolution model (y-axis) is shown for PODs with different costs of infection (s) (30 PODs for each s). Results for single PODs are shown as dots. Note that for these points we added some jitter to the x-values in order to increase the readability of the plots. (TIF) [file pcbi.1007668.s007.tif]

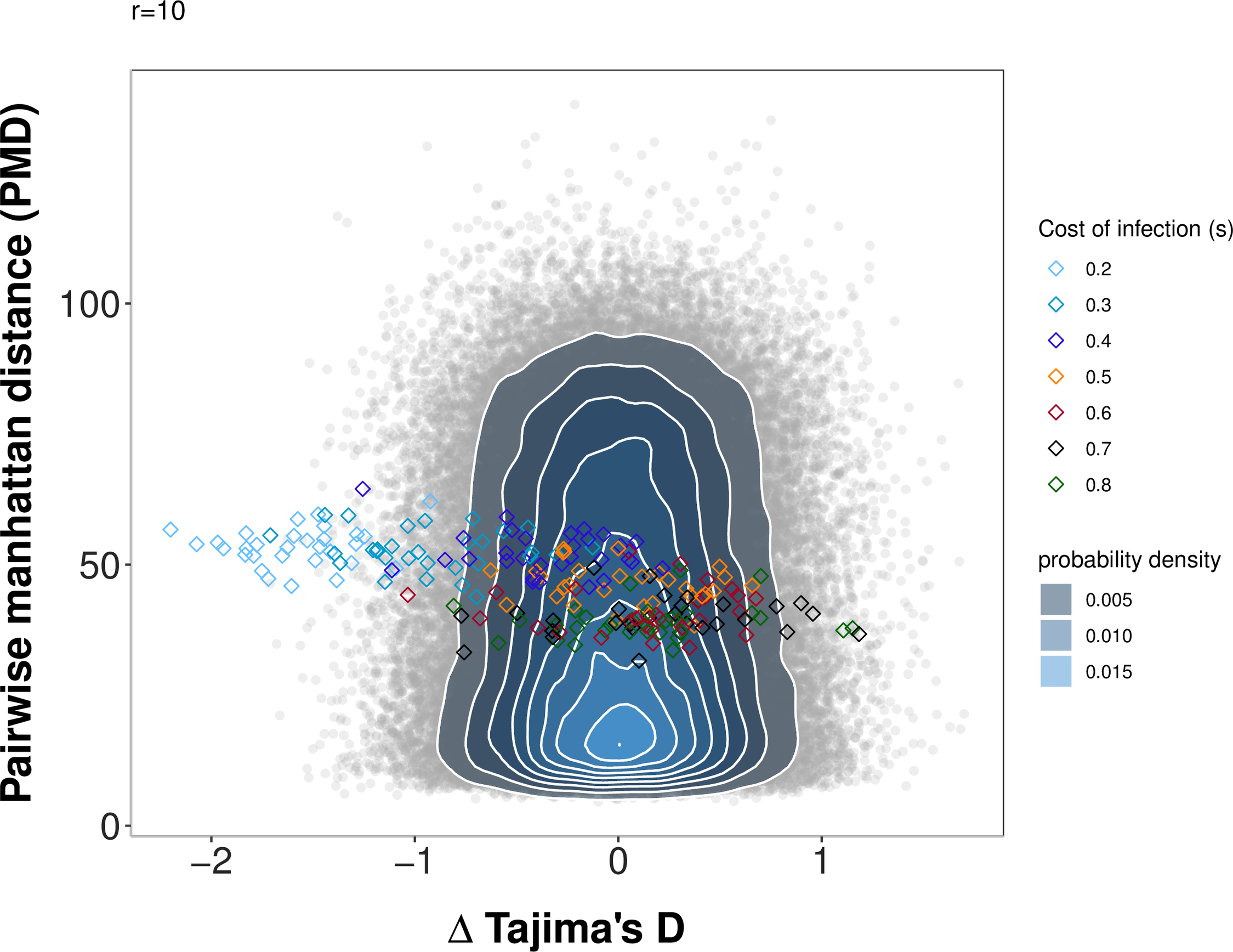

Supplement: S8 Fig — Pairwise Manhattan distance (y-axis) and the difference between Tajima’s D of the host and of the parasite (x-axis) for the PODs used for inference in Scenario 1 and the 100,000 neutral simulations run for this scenario. Under the neutral model, host and parasite population sizes vary. Simulations under the neutral model are shown as grey open circles, and a bivariate normal kernel estimation has been applied to obtain a probability density of the summary statistic combinations. The PODs for scenario 1 are shown as diamonds and are coloured coded based on the true cost of infection (s). (TIF) [file pcbi.1007668.s008.tif]

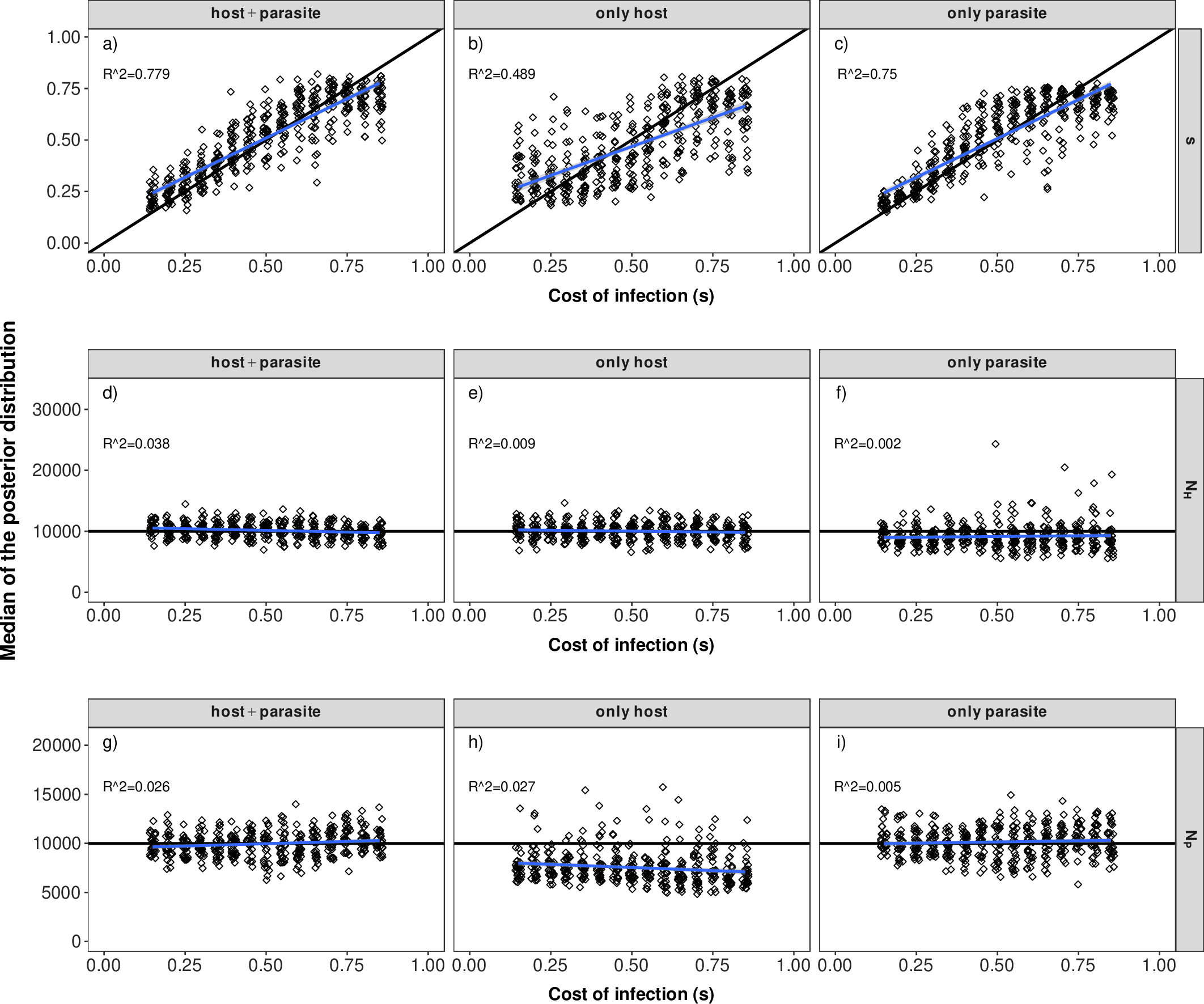

Supplement: S9 Fig — Median of the posterior distribution (y-axis) for the cost of infection s (top, a-c), host population size (NH) (middle, d-f) and parasite population size (NP) (bottom, g-i) when inference is based on host and parasite summary statistics (left), only host summary statistics (middle) or only parasite summary statistics (right) for scenario 1. The median of the posterior distribution (after post-rejection adjustment) is plotted for each POD in scenario 1. The true cost of infection for each POD is shown on the x-axis with jitter added to increase the readability. (TIF) [file pcbi.1007668.s009.tif]

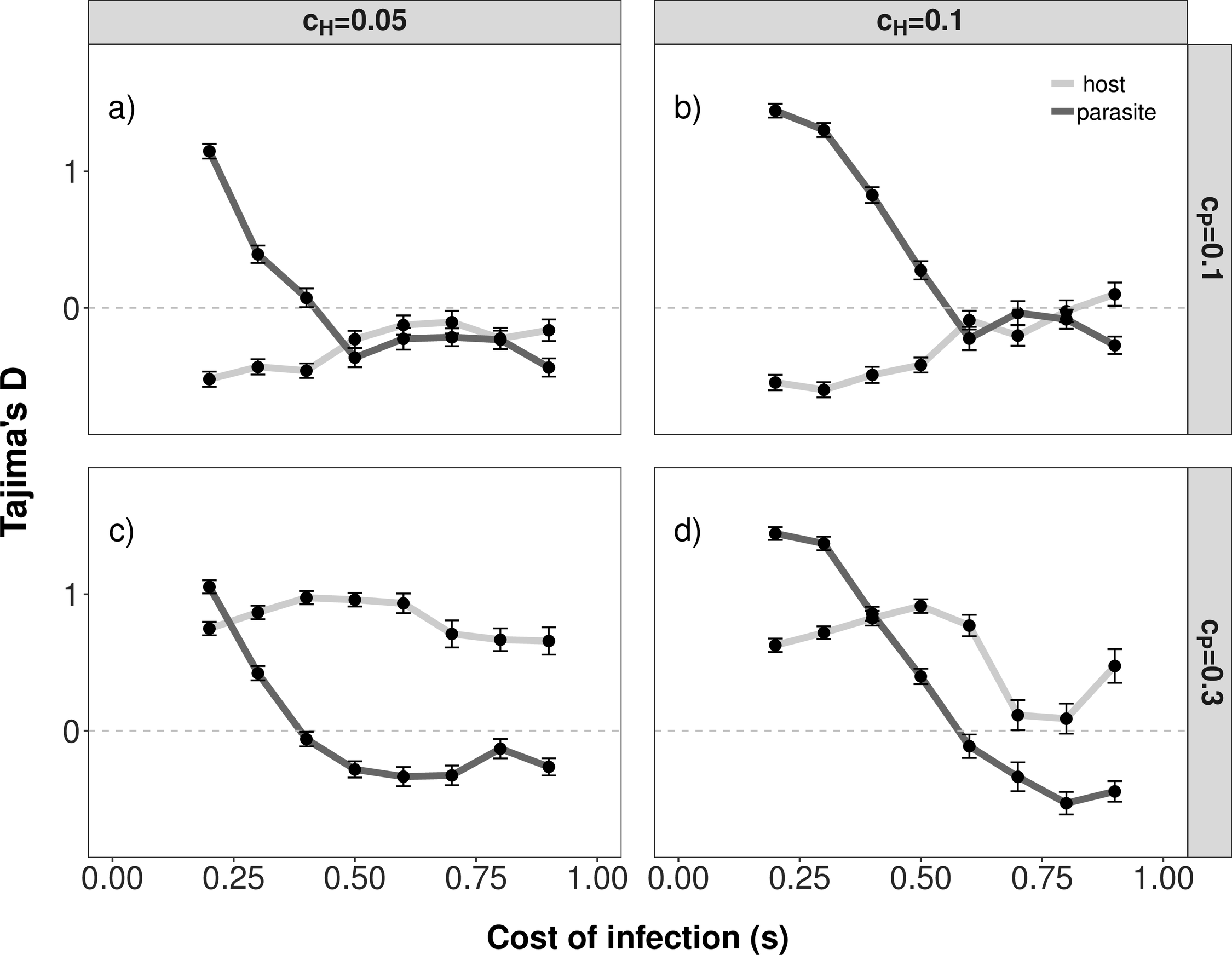

Supplement: S10 Fig — Tajima’s D (y-axis) for model A for various cost of infection s (x-axis). The results are shown for different combinations of cP (cP = 0.1 top, cP = 0.3 bottom) and cH (cH = 0.05 left, cH = 0.1 right). The mean and standard error of Tajima’s D of the parasite population (dark grey) and of the host population (light grey) are plotted for r = 200 repetitions. The dashed-dotted line shows the expected value of Tajima’s D in a Wright-Fisher population with constant population size. Tajima’s ≪ 0 is an indicator of selective sweeps Tajima’s D ≫ 0 is an indicator of balancing selection. The other parameters are fixed to: NH = NP = 10, 000, nH = nP = 50, θH = θP = 5, μRtor = μrtoR = μntoI = μIton = 10−5. (TIF) [file pcbi.1007668.s010.tif]

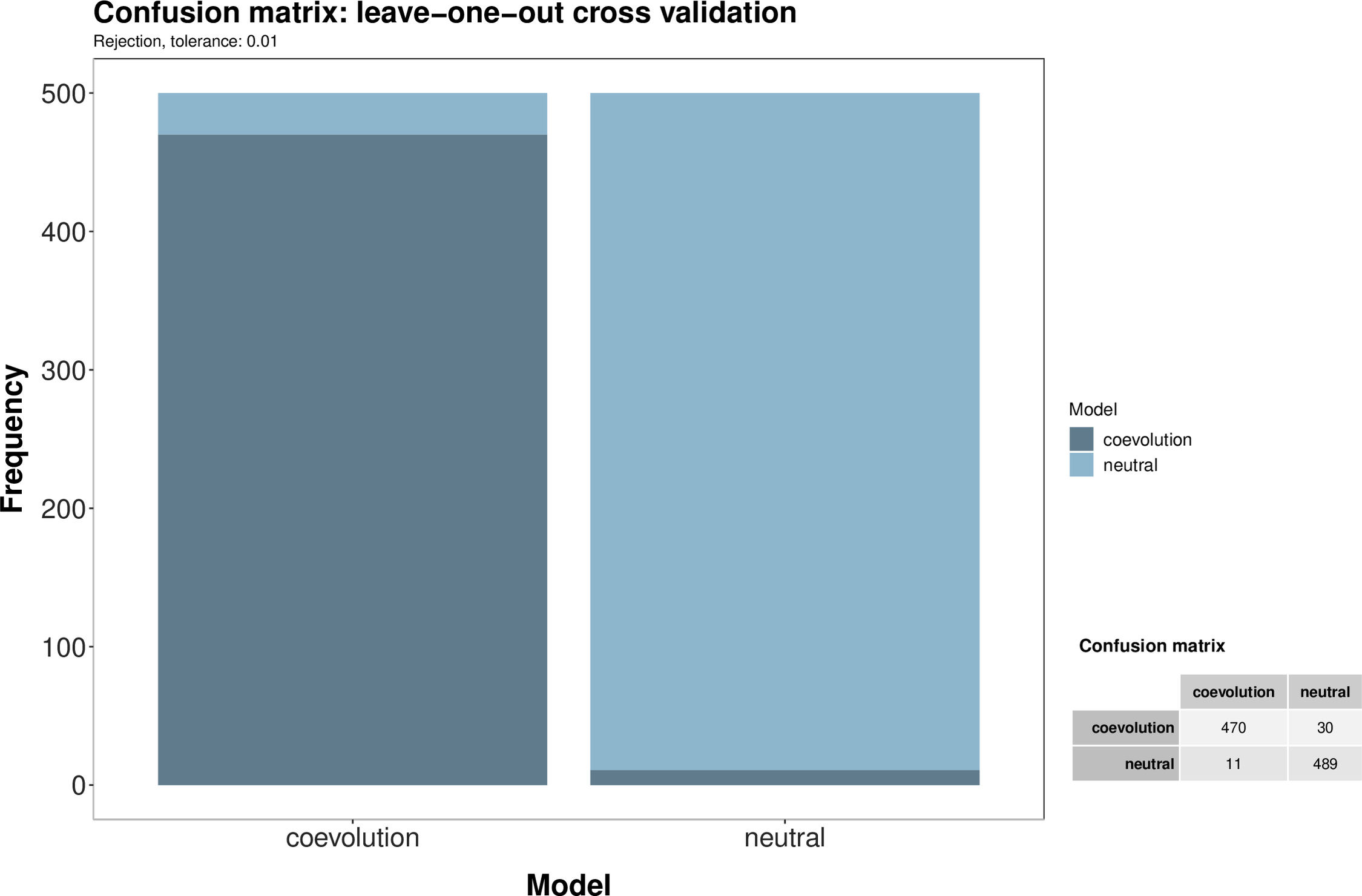

Supplement: S11 Fig — Leave-one-out-cross-validation result for distinguishing the coevolution model with unknown costs of infection (s), cost of resistance (cH) and cost of infectivity (NP) from a neutral model constant host and parasite population sizes (NH = NP = 10, 000). Cross-validation results are shown for r = 30 and are based on 500 randomly chosen ABC-simulations for each model. (TIF) [file pcbi.1007668.s011.tif]

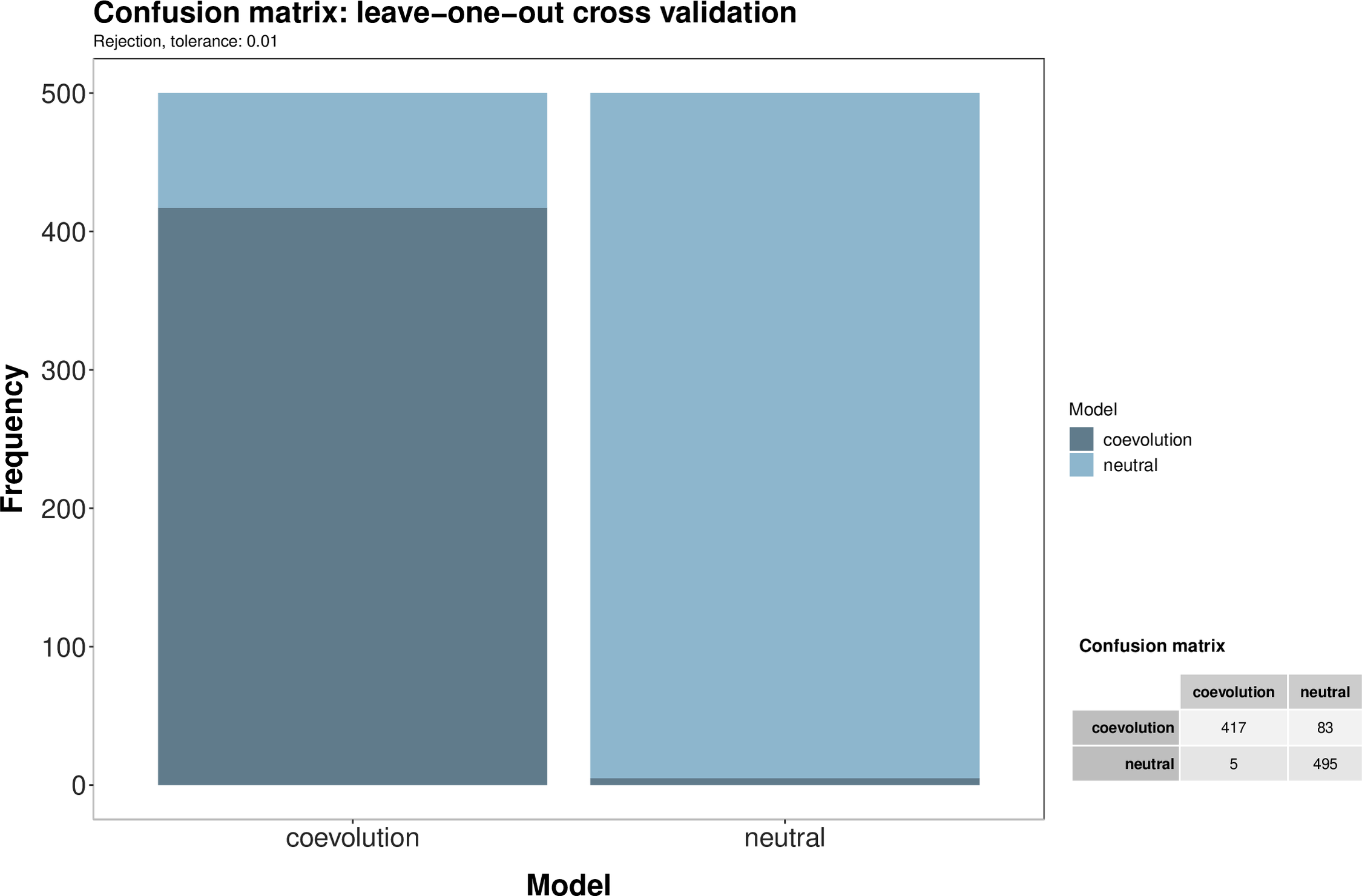

Supplement: S12 Fig — Leave-one-out-cross-validation result for distinguishing the coevolution model with unknown costs of infection (s), cost of resistance (cH) and cost of infectivity (NP) from a neutral model constant host and parasite population sizes (NH = NP = 10, 000). Cross-validation results are shown for r = 10 and are based on 500 randomly chosen ABC-simulations for each model. (TIF) [file pcbi.1007668.s012.tif]

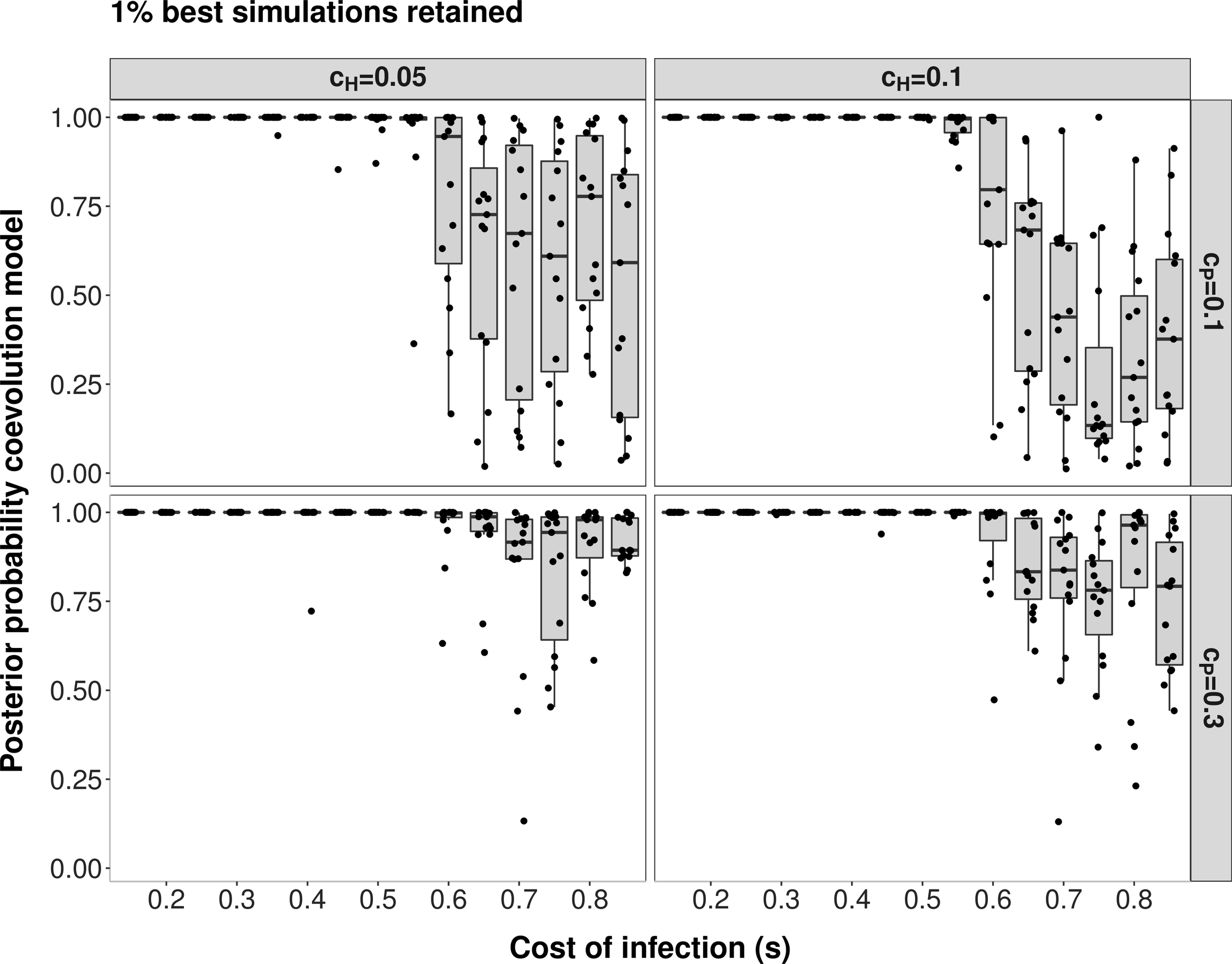

Supplement: S13 Fig — Results are shown for r = 10 and 15 PODs per boxplot. The posterior density in support of the coevolution model (y-axis) is shown for PODs with varying cost of infection (s). The different panels reflect the combination of cH and cP for the respective PODs (left: cH = 0.05, right: cH = 0.1, top: cP = 0.1, bottom: cP = 0.3). Model choice has been run to distinguish a coevolution model with unknown costs of infection (s), cost of resistance (cH) and cost of infectivity (cP) from a neutral model with constant host and parasite population size (NH = NP = 10, 000). Results for single PODs are shown as dots and jitter added to the x-values to increase the readability. (TIF) [file pcbi.1007668.s013.tif]

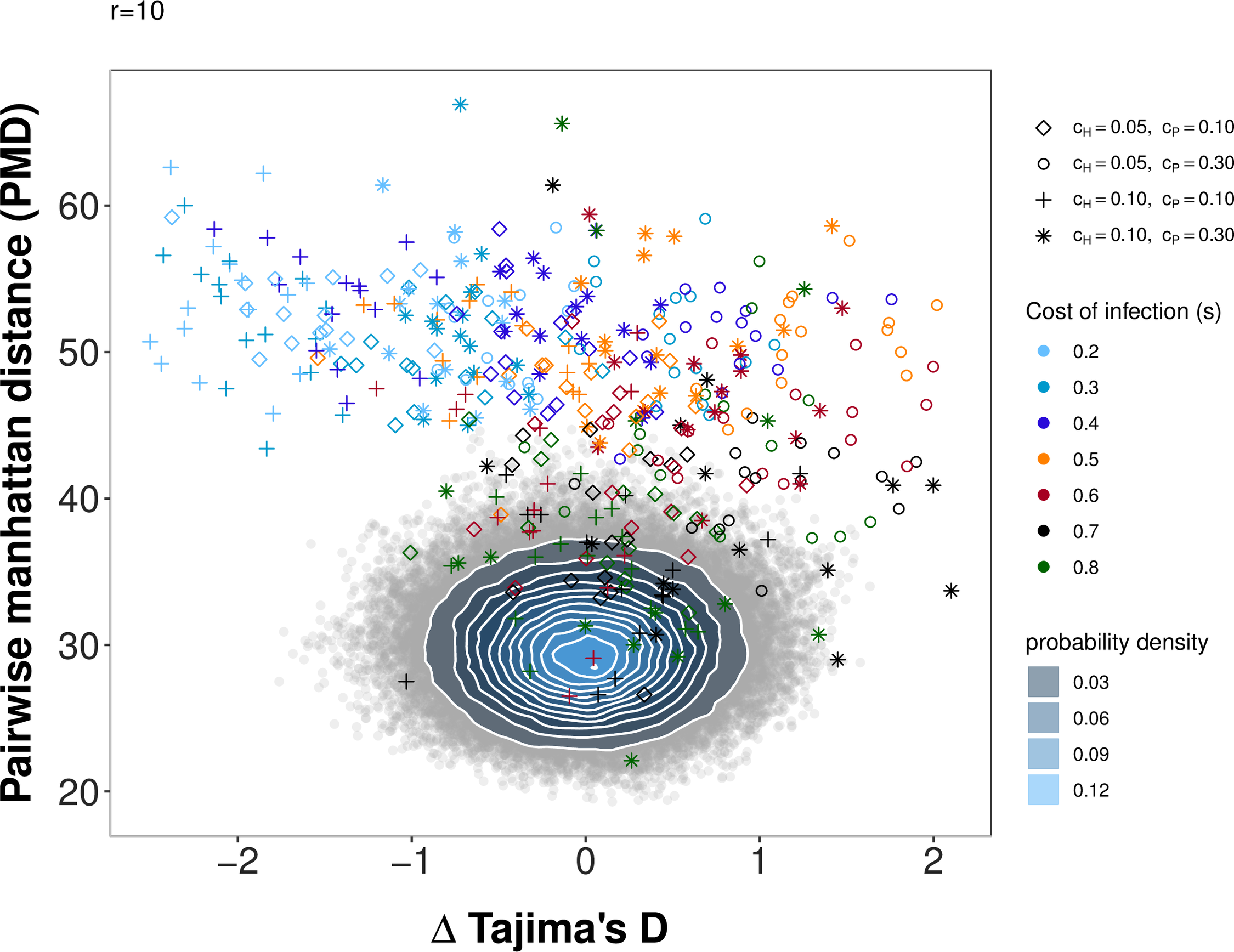

Supplement: S14 Fig — Pairwise Manhattan distance (y-axis) and the difference between Tajima’s D of the host and of the parasite (x-axis) for the PODs used for inference in Scenario 2 and 100,000 neutral simulations. Simulations under the neutral model are shown as grey open circles. A bivariate normal kernel estimation has been applied to obtain a probability density of the different summary statistic combinations. The PODs for scenario 2 are shown in color. Colors reflect the true cost of infection (s) for a particular POD (see legend) and shapes indicate the combination of cH and cP (diamonds: cH = 0.05, cP = 0.1; circles: cH = 0.05, cP = 0.3; crosses: cH = 0.1, cP = 0.1; stars: cH = 0.1, cP = 0.3) for the respective POD. (TIF) [file pcbi.1007668.s014.tif]

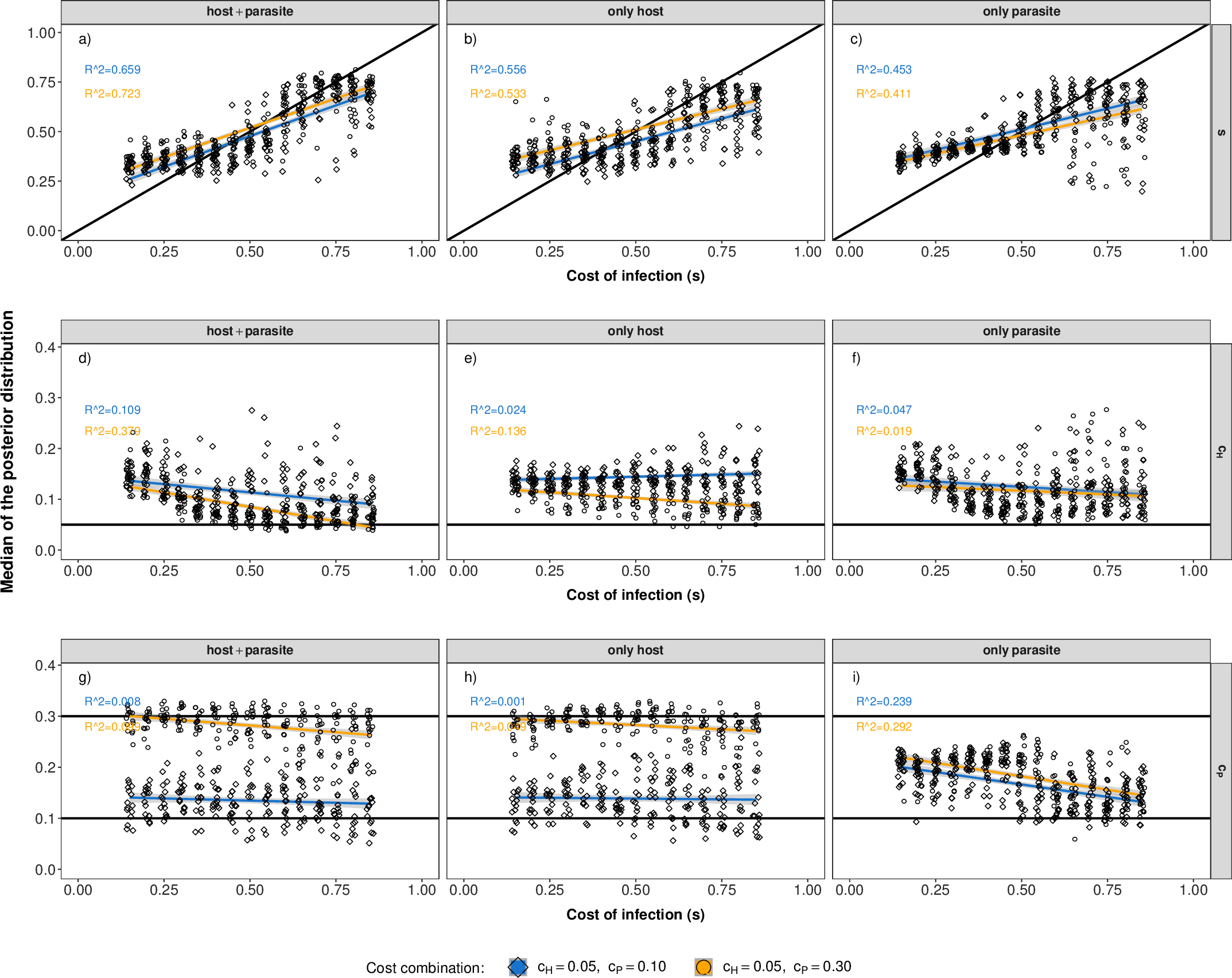

Supplement: S15 Fig — Median of the posterior distribution (y-axis) for the cost of infection s (top, a-c), cost of resistance (cH) (middle, d-f) and cost of infectivity (cP) (bottom, g-i) when inference is based on host and parasite summary statistics (left), only host summary statistics (middle) or only parasite summary statistics (right) for scenario 2. The median of the posterior distribution (after post-rejection adjustment) is plotted for each POD in scenario 2. The true cost of infection for each POD is shown on the x-axis with jitter added to increase the readability. The R2-value of a corresponding linear regression model is shown in each panel. (TIF) [file pcbi.1007668.s015.tif]

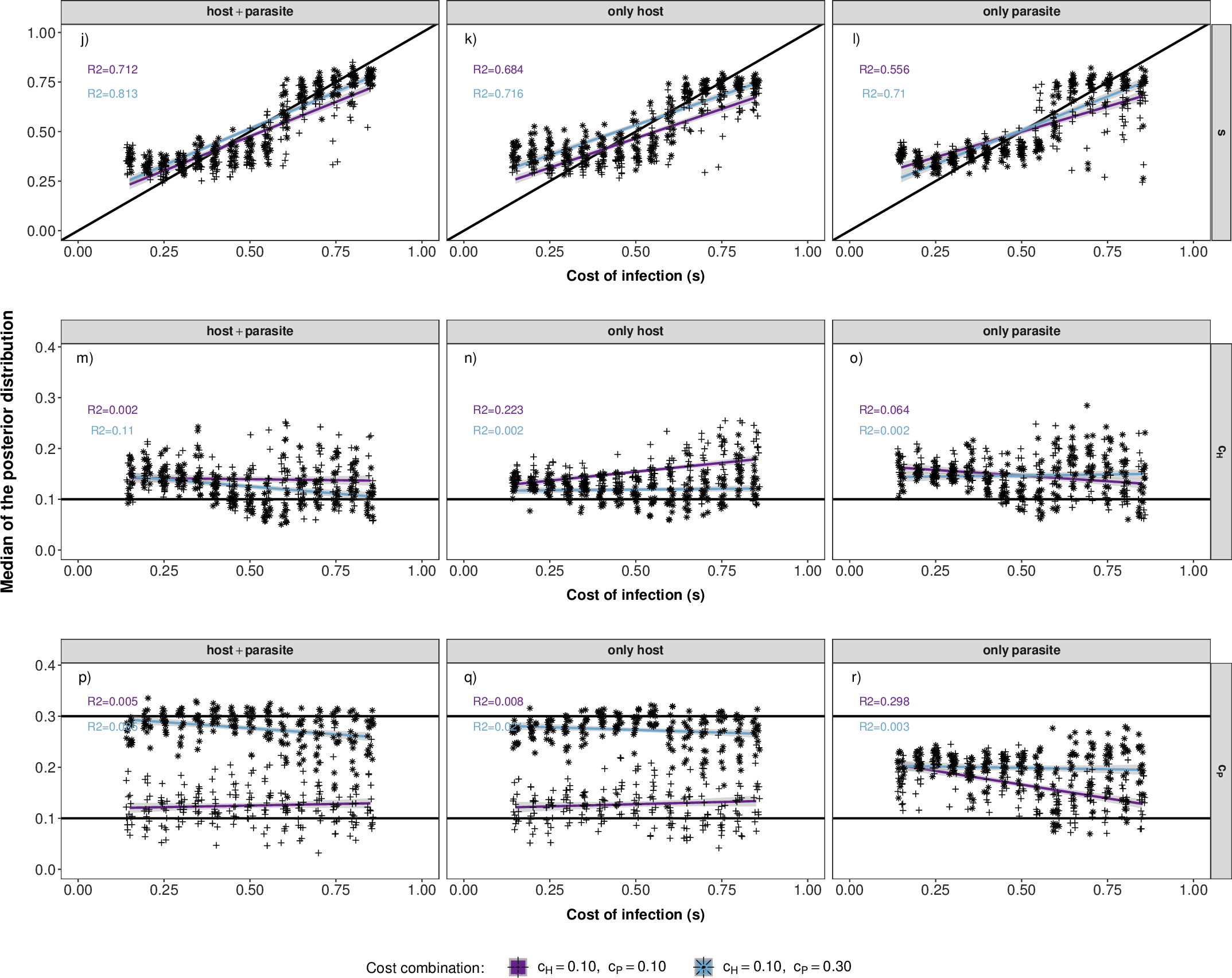

Supplement: S16 Fig — Median of the posterior distribution (y-axis) for the cost of infection s (top, a-c), cost of resistance (cH) (middle, d-f) and cost of infectivity (cP) (bottom, g-i) when inference is based on host and parasite summary statistics (left), only host summary statistics (middle) or only parasite summary statistics (right) for scenario 2. The median of the posterior distribution (after post-rejection adjustment) is plotted for each POD in scenario 2. The true cost of infection for each POD is shown on the x-axis with jitter added to increase the readability. The R2-value of a corresponding linear regression model is shown in each panel. (TIF) [file pcbi.1007668.s016.tif]

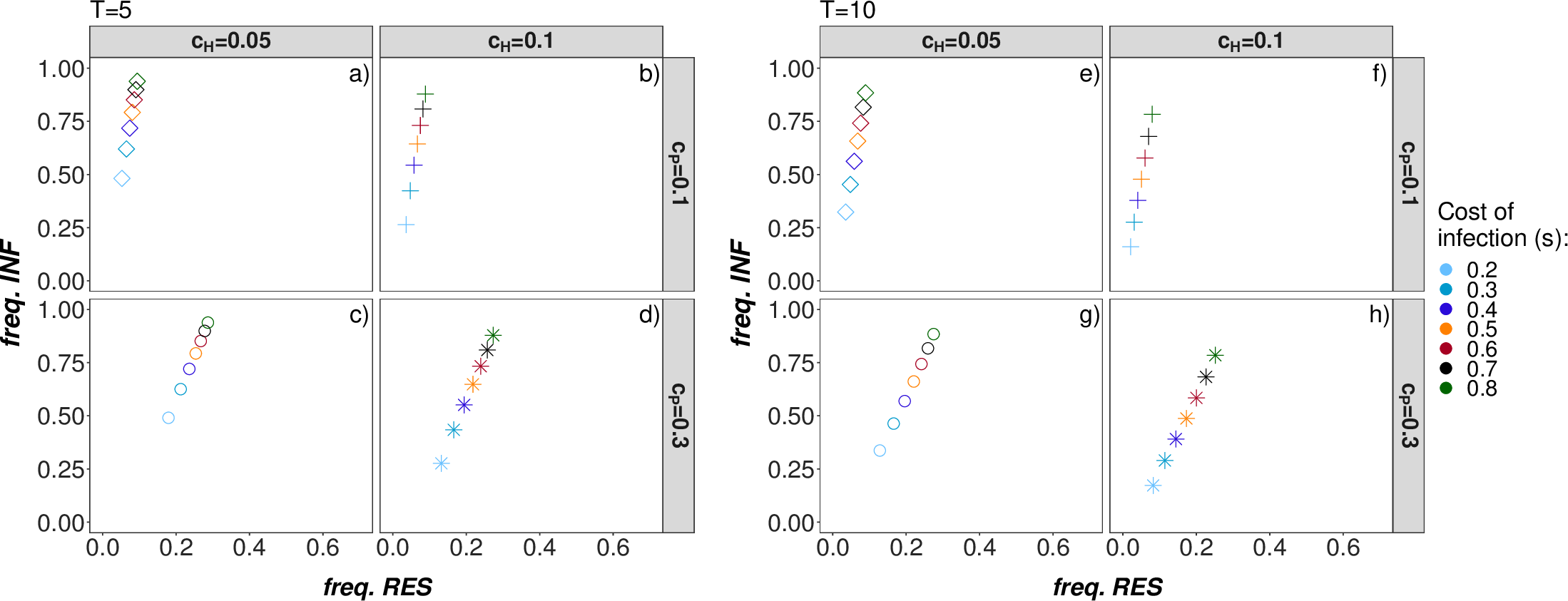

Supplement: S17 Fig — Deterministic equilibrium frequencies for Model B for a) T = 5 parasite generations (left) and b) T = 10 parasite generations (right) per host generation. The equilibrium frequencies for different combinations of cost of resistance cH = (0.05, 0.1) (columns), cost of infectivity cP = (0.1, 0.3) (rows) and cost of infection s = (0.2, 0.3, 0.4, 0.5, 0.6, 0.7, 0.8) (color of the squares) are shown. Only combinations with trench-warfare dynamics are shown. Centres of the squares represent the equilbrium frequencies obtained by simulating numerically the recursion equations in S1 File for gmax = 30, 000 host generations starting with an initial frequency of R0 = 0.2 resistant hosts and a0 = 0.2 infective parasites. (TIF) [file pcbi.1007668.s017.tif]

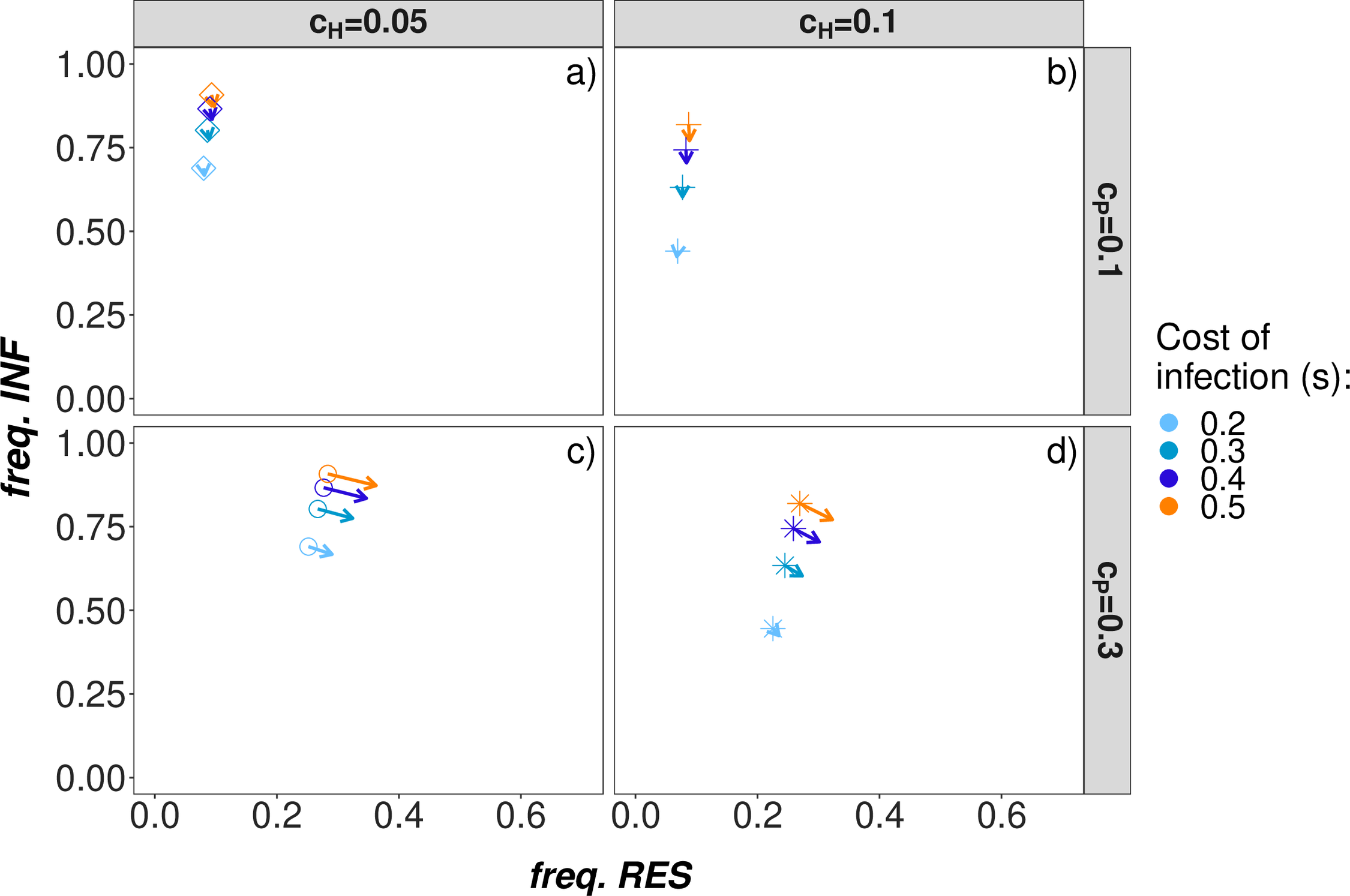

Supplement: S18 Fig — Deterministic equilibrium frequencies for Model C (auto-allo-infection model) with T = 2 parasite generations per host generation and ψ = 0.95. The equilibrium frequencies for different combinations of cost of resistance cH = (0.05, 0.1) (columns), cost of infectivity cP = (0.1, 0.3) (rows) and cost of infection s = (0.2, 0.3, 0.4, 0.5, 0.6, 0.7, 0.8) (color of the squares) are shown. Only combinations which result in trench-warfare dynamics are plotted. Centres of the squares represent the equilbrium frequencies obtained by simulating numerically the recursion equations in S1 File for gmax = 30, 000 host generations starting with an initial frequency of R0 = 0.2 resistant hosts and a0 = 0.2 infective parasites. Heads of the arrows represent the equilibrium frequencies based on Eq (3) which corresponds to the case ψ = 1 [24]. (TIF) [file pcbi.1007668.s018.tif]

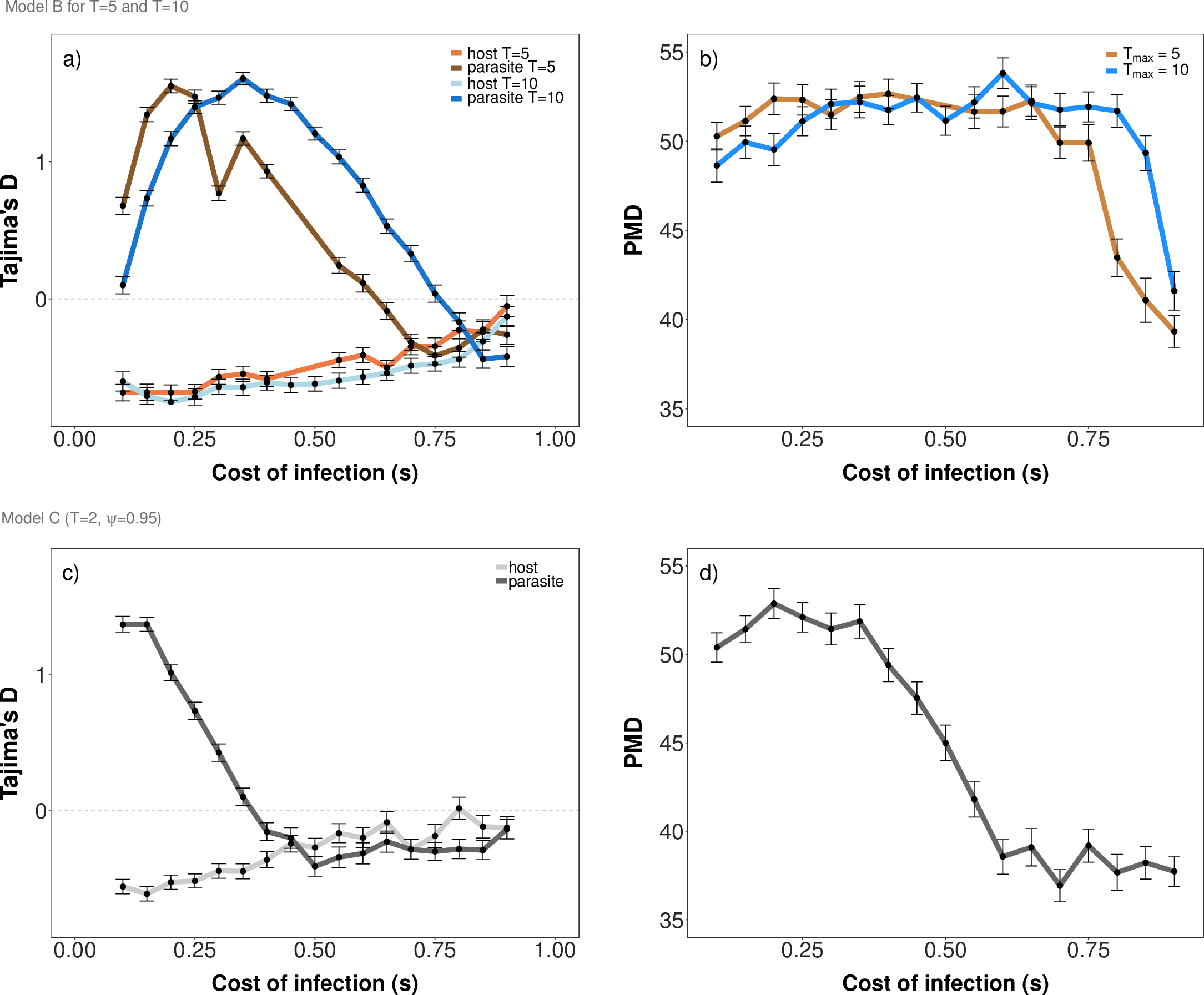

Supplement: S19 Fig — Mean and standard error of Tajima’s D (a+c) and pairwise manhattan distance (PMD) (b+d) for various costs of infection s (x-axis) and r = 200 repetitions. Results for Model B (pure autoinfection model with T = 5 and T = 10) are shown at the top, results for Model C (auto-allo-infection model with ψ = 0.95) are shown at the bottom. The other parameters are fixed to: cH = 0.05 and cP = 0.1. Initial frequencies R0 and a0 in a and b are chosen randomly from a uniform distribution between 0 and 1 while R0 = a0 = 0.2 in c and d. (TIF) [file pcbi.1007668.s019.tif]

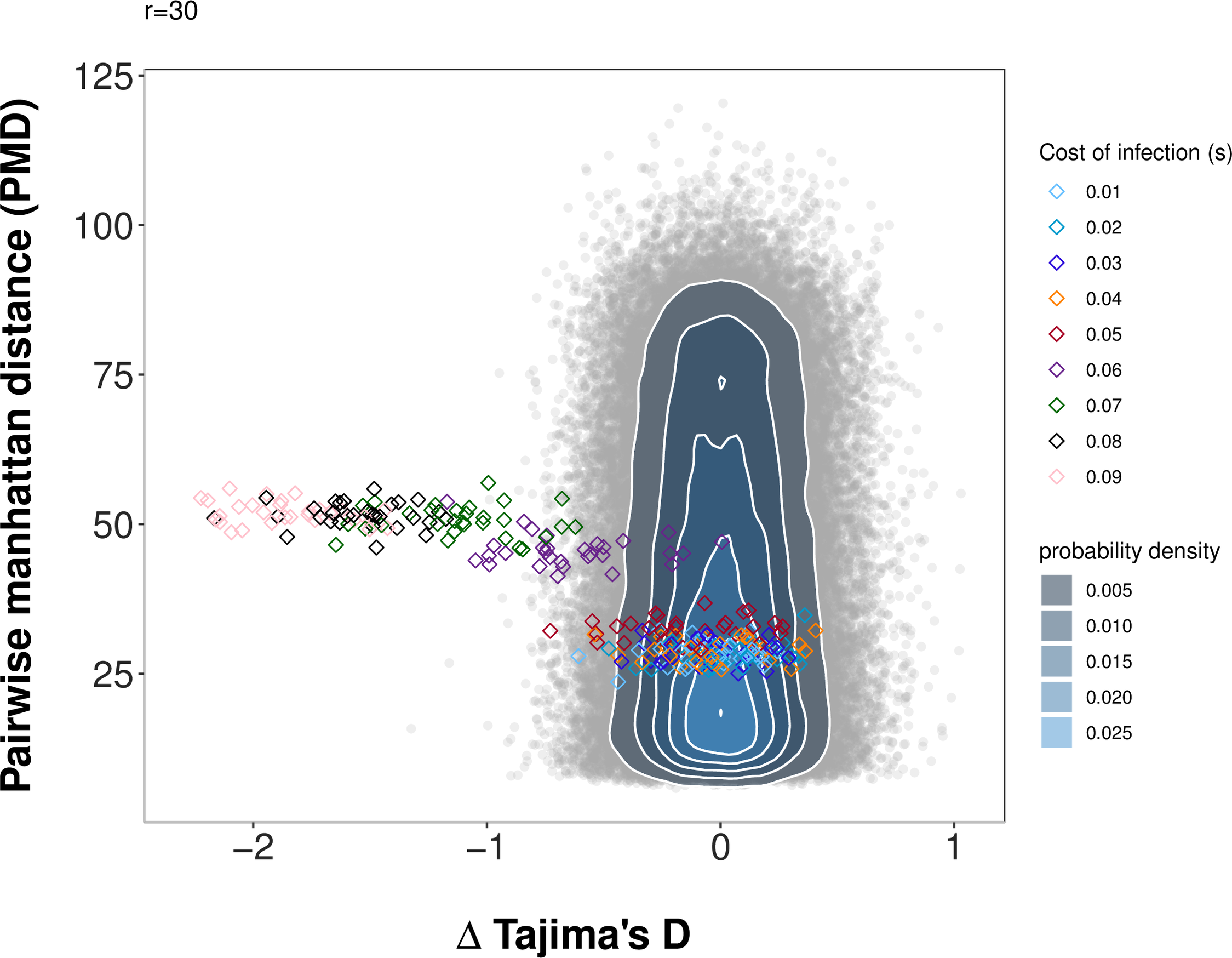

Supplement: S20 Fig — Pairwise Manhattan distance (y-axis) and the difference between Tajima’s D of the host and of the parasite (x-axis) for the PODs used for inference in Scenario 1 and the 100,000 neutral simulations run for this scenario. Under the neutral model, host and parasite population sizes vary. Simulations under the neutral model are shown as grey open circles, and a bivariate normal kernel estimation has been applied to obtain a probability density of the summary statistic combinations. The PODs for scenario 1 are shown as diamonds and are coloured coded based on the true cost of infection (s). (TIF) [file pcbi.1007668.s020.tif]

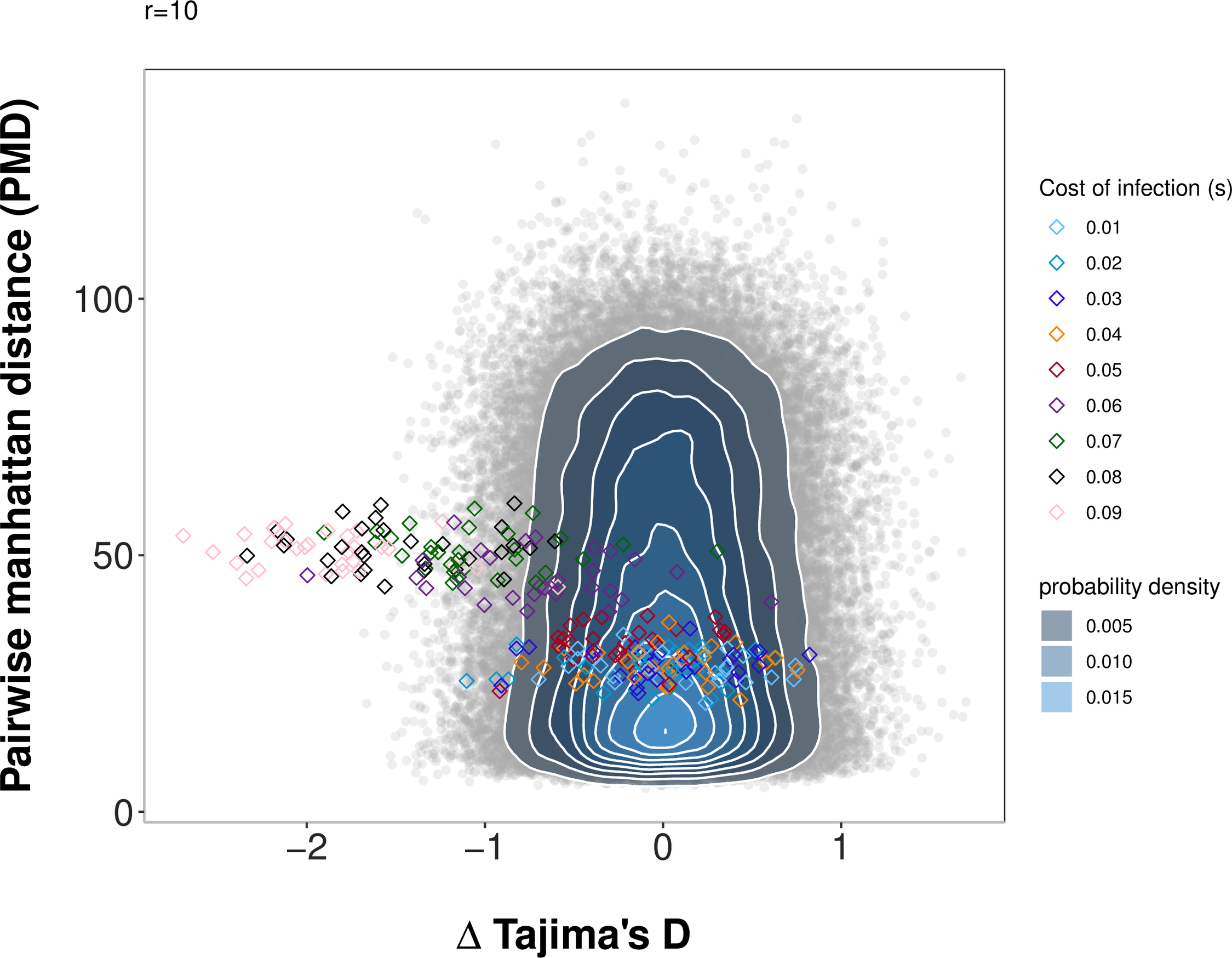

Supplement: S21 Fig — Pairwise Manhattan distance (y-axis) and the difference between Tajima’s D of the host and of the parasite (x-axis) for the PODs used for inference in Scenario 1 and the 100,000 neutral simulations run for this scenario. Under the neutral model, host and parasite population sizes vary. Simulations under the neutral model are shown as grey open circles, and a bivariate normal kernel estimation has been applied to obtain a probability density of the summary statistic combinations. The PODs for scenario 1 are shown as diamonds and are coloured coded based on the true cost of infection (s). (TIF) [file pcbi.1007668.s021.tif]

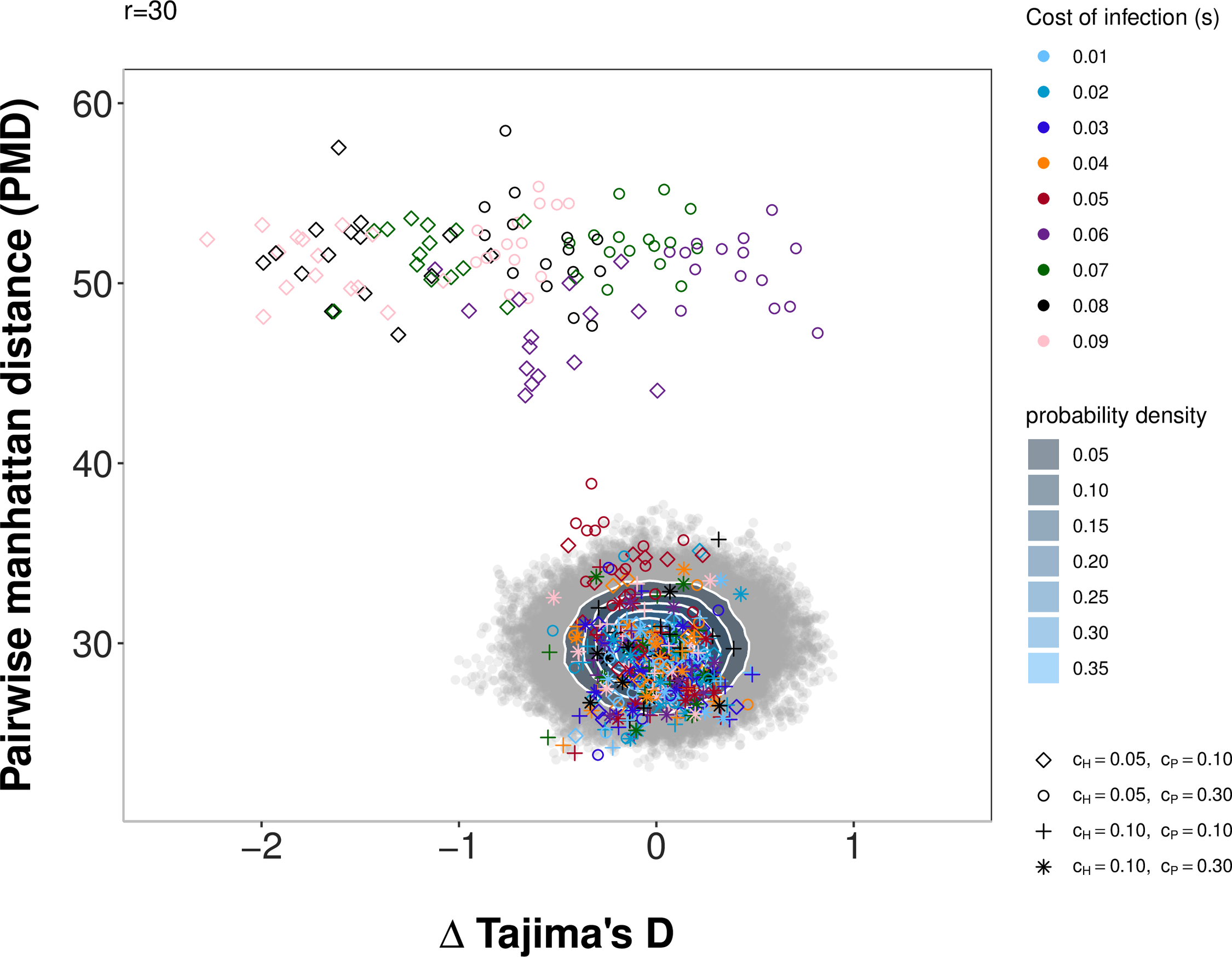

Supplement: S22 Fig — Pairwise Manhattan distance (y-axis) and the difference between Tajima’s D of the host and of the parasite (x-axis) for the PODs used for inference in Scenario 2 and 100,000 neutral simulations. Simulations under the neutral model are shown as grey open circles. A bivariate normal kernel estimation has been applied to obtain a probability density of the different summary statistic combinations. The PODs for scenario 2 are shown in color. Colors reflect the true cost of infection (s) for a particular POD (see legend) and shapes indicate the combination of cH and cP (diamonds: cH = 0.05, cP = 0.1; circles: cH = 0.05, cP = 0.3; crosses: cH = 0.1, cP = 0.1; stars: cH = 0.1, cP = 0.3) for the respective POD. (TIF) [file pcbi.1007668.s022.tif]

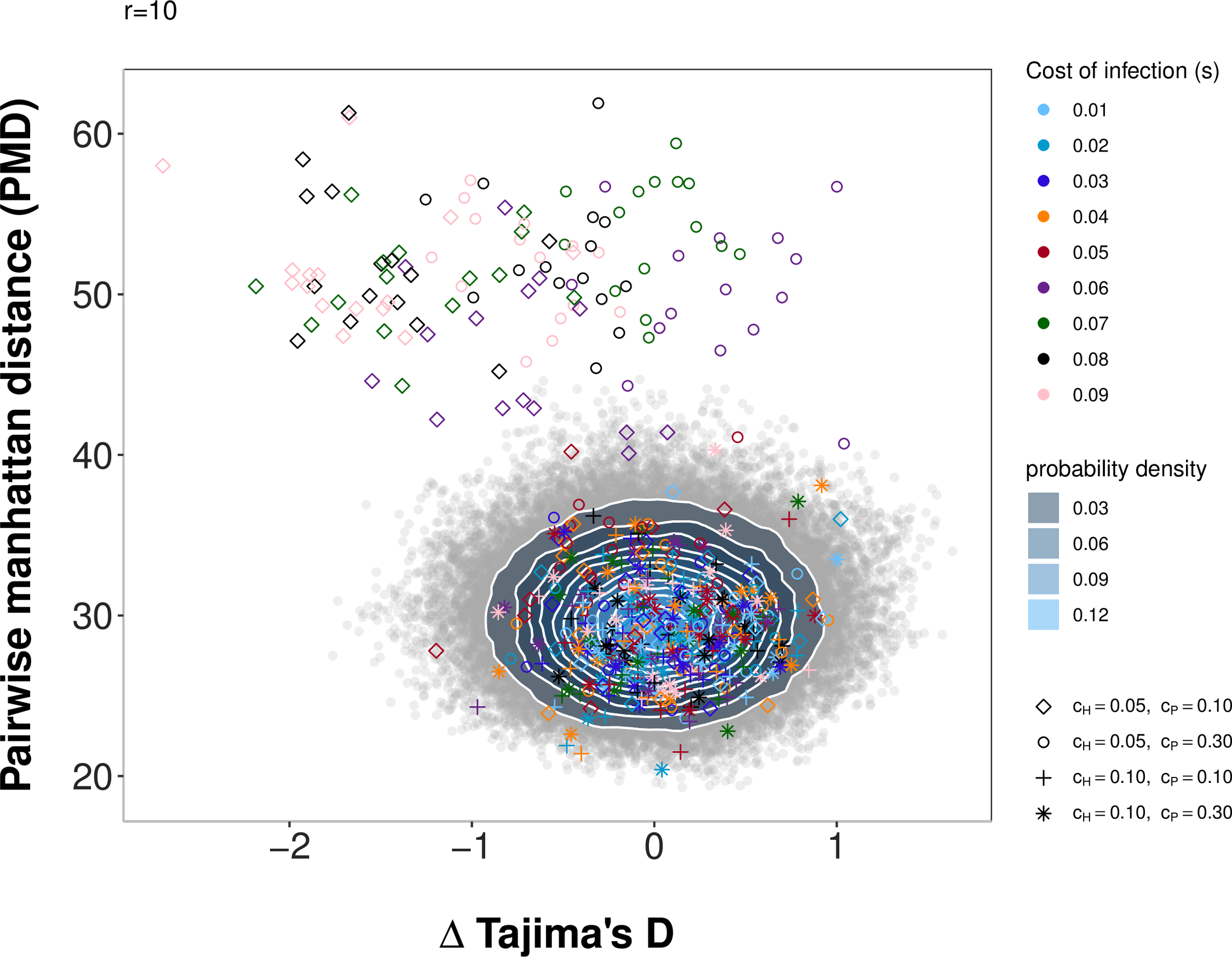

Supplement: S23 Fig — Pairwise Manhattan distance (y-axis) and the difference between Tajima’s D of the host and of the parasite (x-axis) for the PODs used for inference in Scenario 2 and 100,000 neutral simulations. Simulations under the neutral model are shown as grey open circles. A bivariate normal kernel estimation has been applied to obtain a probability density of the different summary statistic combinations. The PODs for scenario 2 are shown in color. Colors reflect the true cost of infection (s) for a particular POD (see legend) and shapes indicate the combination of cH and cP (diamonds: cH = 0.05, cP = 0.1; circles: cH = 0.05, cP = 0.3; crosses: cH = 0.1, cP = 0.1; stars: cH = 0.1, cP = 0.3) for the respective POD. (TIF) [file pcbi.1007668.s023.tif]
