# Supplementary material for: Inference of coevolutionary dynamics and parameters from host and parasite polymorphism data of repeated experiments
Source: PLoS Comput Biol. 2020 Mar 23;16(3):e1007668. doi: 10.1371/journal.pcbi.1007668 (PMC7156111; doi:10.1371/journal.pcbi.1007668)
Supplement: S1 File — (PDF) [file pcbi.1007668.s024.pdf]

Supplementary information coevolution models

Model A

Detailed description how the allele frequency path is obtained

In order to obtain the frequency of a given allele in the next generation, we perform the following steps:

- We compute the frequency *RES*-hosts and *INF*-parasites after selection using the difference equations in Eq (2).
- We incorporate genetic drift by performing a binomial sampling based on these frequencies and the finite and fixed haploid population size (N_H for the host and N_P for the parasite).
- We allow for recurrent allele mutations (functional mutations) to take place and change genotypes from *RES* to *res* at rate μ_{Rtor} or *res* to *RES* at rate μ_{rtoR} in the host and from *ninf* to *INF* at rate μ_{ntoI} and from *INF* to *ninf* at rate $\mu_I ton$ in the parasite. We set all functional mutation rates to $\mu_{Rtor} = \mu_{ntoI} = \mu_{rtoR} = \mu_{Iton} = 10^{-5}$.

Note that the above mentioned steps are repeated twice for the parasite as there are two parasite generation per host generation. Once when going from parasite generation $g,1$ to $g,2$ and once when going from parasite generation $g,2$ to $g+1,1$.
Accordingly, the detailed calculations for each parasite generation are as follows:

1. The expected frequency of *INF*-parasites after selection a_x (x=g,2 or x=g+1,1) is obtained by using the respective recursion equation in Eq. 2. The corresponding frequency of *ninf*-parasites is calculated as $A_x = 1 - a_x$.
2. The number of *INF*-parasite individuals after drift N_I is sampled from a Binomial distribution $N_I \sim \mathcal{B}(N_P,\, a_x)$. Thus, the number of *ninf*-parasites after drift ist equal to $N_n = N_P - N_I$.
3. In order to include the functional mutations the following two samplings are performed:

- the number of mutants M_{In} from *INF* to *ninf* is obtained by sampling from a Poisson distribution with rate $\lambda = \mu_{Iton} \cdot N_I$.
- the number of mutants M_{nI} from *ninf* to *INF* is obtained by sampling from a Poisson distribution with rate $\lambda = \mu_{ntoI} \cdot N_n$.

Thus, the number of *INF*-parasites in generation x is given by:

$$N_{x,I} = N_I - M_{In} + M_{nI} \tag{1}$$

And the frequency of *INF*-parasites at the beginning of generation x is equal to:

$$\frac{N_{x,I}}{N_P} \tag{2}$$

The corresponding steps for the host population are as follows.

1. The expected frequency of *RES*-hosts after selection R_{g+1} is obtained by using difference equation Eq. 2. The frequency of *res*-hosts is calculated as $r_{g+1} = 1 - R_{g+1}$.

2. The number of *RES*-host individuals after drift is sampled from a Binomial distribution $N_R \sim \mathcal{B}(N_H, R_{g+1})$. Thus, the number of *res*-host after drift ist equal to $N_r = N_H - N_R$.

3. In order to include the functional mutations the following two samplings are performed:

   - the number of mutants from *RES* to *res* M_{Rr} is obtained by sampling from a Poisson distribution with rate $\lambda = \mu_{Rtor} \cdot N_R$.
   - the number of mutants from *res* to *RES* M_{rR} is obtained by sampling from a Poisson distribution with rate $\lambda = \mu_{rtoR} \cdot N_r$.

Thus, the number of *RES*-individuals in generation $g + 1$ is given by:

$$N_{g+1,R} = N_R - M_{Rr} + M_{rR} \tag{3}$$

And the frequency of *RES*-hosts at the beginning of generation $g+1$ is equal to:

$$\frac{N_{g+1,R}}{N_H} \tag{4}$$

Model B

In model **Model B** we extend the basic model to $T > 2$ parasite generations per host generation. As in the basic model the cost of infection s_t is a function of the parasite generation t in which the host became infected and the maximum cost of infection s, which correspond to the cost of being infected in the first parasite generation t=1 within host generation g. Upon infection a host stays infected until it reproduces and dies from natural death (at the end of the host generation g). An infected host is reinfected by the offspring of the particular parasite for all subsequent parasite generations within host generation g (100% auto-infection). Hosts which have not been infected so far can be attacked by the offspring of any parasite type at the beginning of each parasite generation t. Whether this interaction subsequently results in an infection depends on the infection matrix. The recursion equations for this model are given by:

$$a_{g,t+1} = \frac{(1-c_P)\left[a_{g,1} + \sum_{l=2}^{t} a_{g,l} R_g \prod_{m=1}^{l-1} A_{g,m}\right]}{(1-c_P)\left[a_{g,1} + \sum_{l=2}^{t} a_{g,l} R_g \prod_{m=1}^{l-1} A_{g,m}\right] + A_{g,1} r_g} \tag{5a}$$

$$a_{g+1,1} = \frac{(1-c_P)\left[a_{g,1} + \sum_{l=2}^{T} a_{g,l} R_g \prod_{m=1}^{l-1} A_{g,m}\right]}{(1-c_P)\left[a_{g,1} + \sum_{l=2}^{T} a_{g,l} R_g \prod_{m=1}^{l-1} A_{g,m}\right] + A_{g,1} r_g} \tag{5b}$$

$$a_{g,2} = \frac{(1-c_P)\cdot a_{g,1}}{(1-c_P)a_{g,1} + A_{g,1} r_g} \tag{5c}$$

$$R_{g+1} = \frac{R_g \cdot (1-c_H)\left((1-s_1)\,a_{g,1} + \sum_{t=2}^{T}\left((1-s_t)a_{g,t}\prod_{l=1}^{t-1} A_{g,l}\right) + \prod_{t=1}^{T} A_{g,t}\right)}{R_g \cdot (1-c_H)\left((1-s_1)\,a_{g,1} + \sum_{t=2}^{T}\left((1-s_t)a_{g,t}\prod_{l=1}^{t-1} A_{g,l}\right) + \prod_{t=1}^{T} A_{g,t}\right) + r_g(1-s_1)} \tag{5d}$$

$A_{g,t}$ ($a_{g,t}$) denotes the frequency of *ninf* (*INF*)-parasites in the t-th parasite generation within host generation g. R_g (r_g) denotes the frequency of *RES* (*res*)-hosts in host generation g.

Note that in this side analysis, genetic drift and functional mutations are only taken into account when going from host generation g to host generation $g + 1$ in both, the host and the parasite. The frequency path in the parasite which is used to launch msms consists of the frequencies at the first parasite generation within host generation g. Time is rescaled as $g_P^* = g/(2N_P)$ in the parasite.

Model C

Model C is based on Model C in [1]. As in model A, we assume $T = 2$ discrete parasite generations per discrete host generation g and frequency-dependent disease transmission. Parasites of the second ($t = 2$) generation within host generation g infect the same host individual as there parent at rate ψ (auto-infection) or a different host at rate $1-\psi$ (allo-infection). A host which is infected throughout the whole host generation g looses the amount $s_1 = s$ (cost of infection) of its fitness. If it is only infected during a single parasite generation the cost of infection reduces to $s_2 = s/2$. The equations of the model can be written as:

$$a_{g,2} = \frac{a_{g,1} \cdot (1 - c_P)}{a_{g,1} \cdot (1 - c_P) + A_{g,1} \cdot r_g} \tag{6a}$$

$$a_{g+1,1} = \frac{(1 - c_P) \cdot (R_g A_{g,1} a_{g,2} + r_g A_{g,1} a_{g,2}(1 - \psi) + a_{g,1}[\psi + a_{g,2}(1 - \psi)])}{r_g \cdot (\psi A_{g,1} + A_{g,2}(1 - \psi)) + (1 - c_P) \cdot (R_g A_{g,1} a_{g,2} + r_g A_{g,1} a_{g,2}(1 - \psi) + a_{g,1}[\psi + a_{g,2}(1 - \psi)])} \tag{6b}$$

$$R_{g+1} = \frac{R_g \cdot (1 - c_H)(A_{g,1} A_{g,2} + (1 - s_2)(A_{g,1} a_{g,2} + a_{g,1} A_{g,2}(1 - \psi)) + (1 - s_1)(a_{g,1}\psi + a_{g,1} a_{g,2}(1 - \psi)))}{R_g \cdot (1 - c_H)\,(A_{g,1} A_{g,2} + (1 - s_2)(A_{g,1} a_{g,2} + a_{g,1} A_{g,2}(1 - \psi)) + (1 - s_1)(a_{g,1}\psi + a_{g,1} a_{g,2}(1 - \psi))) + r_g(1 - s_1)} \tag{6c}$$

$A_{g,t}$ ($a_{g,t}$) denotes the frequency of *ninf* (*INF*)-parasites in the t-th parasite generation within host generation g. R_g (r_g) denotes the frequency of *RES* (*res*)-hosts in host generation g. The allele frequency path for this model is obtained in the same way as for **Model A**.

References

[1] Tellier A, Brown JKM. Stability of genetic polymorphism in host-parasite interactions. P Roy Soc B-Biol Sci. 2007;274(1611):809–817. doi:10.1098/rspb.2006.0281.
