# Supplementary material for: Inference of coevolutionary dynamics and parameters from host and parasite polymorphism data of repeated experiments
Source: PLoS Comput Biol. 2020 Mar 23;16(3):e1007668. doi: 10.1371/journal.pcbi.1007668 (PMC7156111; doi:10.1371/journal.pcbi.1007668)
Supplement: S2 File — (PDF) [file pcbi.1007668.s025.pdf]

Supplementary information Pairwise Manhattan Distance

PMD is calculated as the sum of manhattan distances between class i in the host site frequency spectrum and class i in the parasite site frequency spectrum. It is calculated as:

$$PMD = \sum_{i=1}^{n-1} |\xi_{H,i} - \xi_{P,i}| \quad (1)$$

with $\xi_{H,i}$ ($\xi_{P,i}$) being the total number of neutral SNPs linked to the coevolving locus which are in frequency class i of the unfolded site frequency spectrum of the host (parasite). Note that in the current formulation the summary statistic relies on the sample size of the host (n_H) and the parasite (n_P) being the same. However it is possible to adjust this summary statistic by downsampling the site frequency spectrum of the species with the higher sample size.
